# An open source statistical web application for validation and analysis of virtual cohorts

**DOI:** 10.1038/s41598-025-99720-3

**Published:** 2025-05-06

**Authors:** Christian Ohmann, Takoua Khorchani, Alexandru Cracanel, Jan Brüning, Pablo Emilio Verde

**Affiliations:** 1 European Clinical Research Infrastructures Network (ECRIN), Kaiserswerther, Strasse 70, 40477 Düsseldorf, Germany; 2 European Clinical Research Infrastructure Network (ECRIN), 30 Bd Saint-Jacques, 75014 Paris, France; 3 Automation and Information Technology, Transilvania University of Brasov, Mihai Viteazu nr. 5, 5000174 Brasov, Romania; 4 Institut Für Kardiovaskuläre Computer-Assistierte Medizin, Charité - Universitätsmedizin Berlin, Augustenburger Pl. 1, 13353 Berlin, Germany; 5 Coordination Centre for Clinical Trials, Heinrich Heine University Düsseldorf, Moorenstrasse 5, 40225 Düsseldorf, Nordrhein-Westfalen Germany

**Keywords:** Virtual cohort, In-silico trial, Web application, Validation, Shiny, SIMCor, Biotechnology, Computational biology and bioinformatics, Medical research

## Abstract

The conventional approach to developing medical treatments and medical devices usually covers pre-clinical and in-vitro investigations, in-vivo animal studies and clinical trials with humans. In-silico trials and virtual cohorts present a promising avenue for addressing the challenges inherent in clinical research and improving its efficiency. Despite considerable advancements in the field of in-silico trials, several notable gaps and challenges still need to be addressed, one is the limited availability of open and user-friendly statistical tools to support the specific analysis of virtual cohorts and in-silico trials. In the EU-Horizon funded project SIMCor we have developed a web application, providing a R-statistical environment supporting the validation of virtual cohorts and the application of validated cohorts for in-silico trials. It provides a practical platform for validating cohorts and has implemented existing statistical techniques that can be applied to compare virtual cohorts with real datasets. It is fully open, generic and menu driven and provides user guidance and help (https://github.com/ecrin-github/SIMCor, https://zenodo.org/records/14718597).The tool has been developed according to specified user requirements and has been extensively tested and validated. Important next steps are to gain more experience with the tool in other domains and research environments and to extend its functionality.

**Supplementary Information:**

The online version contains supplementary material available at 10.1038/s41598-025-99720-3.

## Introduction

An in-silico clinical trial, also known as a virtual clinical trial, is an individualised computer simulation used in the development or regulatory evaluation of a medicinal product, device, or intervention^1,2^. The conventional approach to developing medical treatments and devices typically commences with pre-clinical and in-vitro development, followed by in-vivo animal models, and then clinical trials to evaluate the product’s viability for human use. In-silico trials and virtual cohorts, which are de-identified virtual representations of real patient cohorts, present a promising avenue for addressing the challenges inherent in clinical research, such as long durations, high costs, as well as ethical implications, and improving its efficiency. Literature reports suggest that in-silico clinical trials can help to reduce, refine, and partially replace real clinical trials by reducing their size and duration through better design, refining clinical trials with clearer, and more detailed information on potential outcomes, by enhancing the understanding of how the tested product works^3–5^. Under appropriate conditions, in-silico trials can also partially replace clinical trials^6^. In addition, modelling and simulation is used to reduce, refine and replace animal experimentation and even to replace bench tests^2^. However, it must be noted that full adoption of this potential will also require changes in legislation next to efforts in academic and industrial research, as animal trials are, to this day, a fundamental aspect of the pathway towards regulatory approval of medical devices. That this full potential can be achieved has been demonstrated by the wider application of in-silico clinical trials in pharmacological research, where regulatory acceptance of this novel technology is already strong^7^. Here, in-silico trials have been shown to be able to predict toxicity and safety and even being more efficient than animal trials^1^.

Furthermore, in-silico trials considerable time and cost savings can be achieved. This was exemplarily demonstrated by the VICTRE study, where only one third of the resources were required to design and complete a comparative trial. The comparative trial took approximately 4 years to complete, versus 1.75 years for the design, implementation, and execution of the VICTRE trial^8^. Similarly, the FD-PASS trial investigating safety and efficacy of flow diverter devices has been successfully replicated using in-silico models. In addition to the above-mentioned benefits, the respective study found, that the in-silico trial was able to provide more information and insights regarding treatment failure than its conventional counterpart^5^. Currently, proper quantification of potential cost savings is challenging as information regarding costs of clinical trials are often confidential. However, the demonstrated effect of time saving and therefore potentially reducing the time to market is hugely beneficial, as it will ultimately result in earlier generation of revenue, which is considered largely beneficial to the medical device industry^9^.

Despite the promising advancements in the field of in-silico trials, several notable gaps and challenges still need to be addressed, hindering their wider adoption in healthcare and clinical research. These include technological limitations and advances, need for clarity on model evaluation, the unmet need for regulatory guidance, and poor communication between stakeholders.

For performing in-silico trials, adequate and powerful statistical tools are needed for planning and analysis. Several toolboxes and platforms are currently available. Some of them are commercial (e.g., InSilico trial platform with many services to support drug development^10^), while others are open-source and (partially) freely available (e.g., The QSP (quantitative systems pharmacology) Toolbox^11^, Universal Immune System Simulator (UISS)^12^, Simulo^13^). In addition, clinical trial simulators, such as the Highly Efficient Clinical Trials Simulator (HECT), may be valuable for designing in-silico trials^14^. While these tools are promising, certain limitations could reduce their applicability and usefulness.

The importance of virtual cohorts and in-silico techniques was investigated in the SIMCor project. SIMCor is a three-and-a-half year (January 2021–June 2024) EU-Horizon 2020 research and innovation action developing a computational platform for in-silico development, validation and regulatory approval of cardiovascular implantable devices^15^. The platform, composed of a virtual cohort generation and validation domain, a device implantation and effect simulation domain, and equipped with a variety of in-silico modelling resources, represents an open environment for collaborative R&D (research & development) among device manufacturers, researchers, medical authorities and regulatory bodies. In the project, in-silico technologies are applied to two clinical use cases: the simulation of a Transcatheter Aortic Valve Implantation (TAVI), and a Pulmonary Arterial Pressure Sensor (PAPS)^16^.

In this project, a general methodological framework for assessing the impact of in-silico methods and technologies on clinical and preclinical trials was developed. An essential part of this framework involves a statistical environment for planning and analysing virtual cohorts and in-silico trials. After careful evaluation, it was concluded that existing tools and platforms only partly fulfill the needs of the project, leading to the decision to develop and implement a more generic and open statistical environment for analysis of in-silico trials. The tool is based on multi-functionality, adequate software and hardware architecture, and high levels of automation. It is designed to support the use cases in SIMCor but also to be applicable to other use cases and domains. This paper is dedicated to the development, testing/validation, and application of this tool in the SIMCor project.

## Methods

### Survey on existing tools

To prepare the development of the R-Statistical environment for application in virtual cohorts and in-silico trials, a survey about existing R-tools for computational modelling has been performed. Only R-packages that were listed in CRAN (Comprehensive R Archive Network) were included. The search was restricted to R-packages from CRAN for the following reasons:

- Statistical software free available and open source
- Structured and proofed quality management in software development
- Flexible integration into low level computer languages (e.g., C +  + , Fortran) and scripting languages for data science (e.g., Python, Mathlab, Julia)
- Support of graphical user interfaces like web applications
- Scalability to dynamically implement further complex statistical procedures into the software

The search was performed in October 2023 with the list of CRAN packages by name (https://cran.r-project.org/web/packages/available_packages_by_name.html). CRAN packages referring to “clinical trials” (respectively “platform trials”, “adaptive trials” as subcategory) were filtered (n = 94) and then manually searched with respect to “simulation” in the title. In total, 8 applications could be identified, as relevant for computational modelling (supplementary material S1). The packages were assessed and information consented with respect to name, short description, URL, open source, licence and target by two co-authors of the manuscript (CO, PEV). Close consideration of the existing tools revealed that currently no R-package exists that fully covers the needs for analysing data related to virtual cohorts and in-silico trials. These needs were discussed in a workshop described below. For that reason, the decision was taken to develop a web application and templates for planning and analysis of in-silico trials, allowing validation of virtual cohorts as well as application of validated cohorts in in-silico trials.

### Statistical environment and requirements

A virtual workshop entitled “Statistical environment for in-silico trials” was organised by SIMCor and took place on 6 December 2021 with around 50 participants, including representatives from other in-silico projects (In-silico World^17^, SimInSitu^18^, SimCardioTest^19^). The objective of the workshop was to discuss and specify the requirements for the implementation of a statistical analysis environment for planning and managing in-silico trials and to explore implementation strategies together with experts from in-silico research as well as experienced biostatisticians, data managers, and data scientists (Supplementary Material S2).

Following the workshop, the decision was taken to develop the statistical environment with R. R-Markdown and Shiny. The combination of R with R Markdown and Shiny packages provides a widely used and user-friendly ecosystem for planning, analysis, and reporting in-silico trials within a replicable research environment. Detailed arguments for using this environment are given in the supplementary material S3 and in the section on “Survey on existing tools”. R-packages in CRAN are issued under the GNU-2 licence. The GNU General Public Licenses (GNU GPL or simply GPL) are a series of widely used free software licenses, or copyleft licenses, that guarantee end users the freedom to run, study, share, and modify the software. In the project, other packages from CRAN have been used, which are under the GNU-2 license. The R-statistical environment developed is also issued under the GNU-2 licence.

In the following the web application developed with Shiny is named R-statistical environment.

Practical software development started with the provision of user stories. User stories are tokens representing “atomic” needs and functionalities expected from the system. It includes information on how the stakeholder describes the steps or workflow to solve each part of the problem addressed. In our project, user stories were defined based on the requirements identified in the initial workshop, and the implementation was done in iterations/sprints, where the development team delivered incremental features and received constant feedback from main stakeholders (Agile methodology, supplementary material S4).

### Statistical algorithms to be implemented

The analytical techniques to be implemented in the R-statistical environment are described in detail in a document “general model” (version 2, December 2023**,** supplementary material S5). An overview is given in the figure below.

Two major areas are covered: validation of virtual cohorts on real datasets and application of validated cohorts in in-silico trials.

## Results

### Implementation

**Fig. 1:**
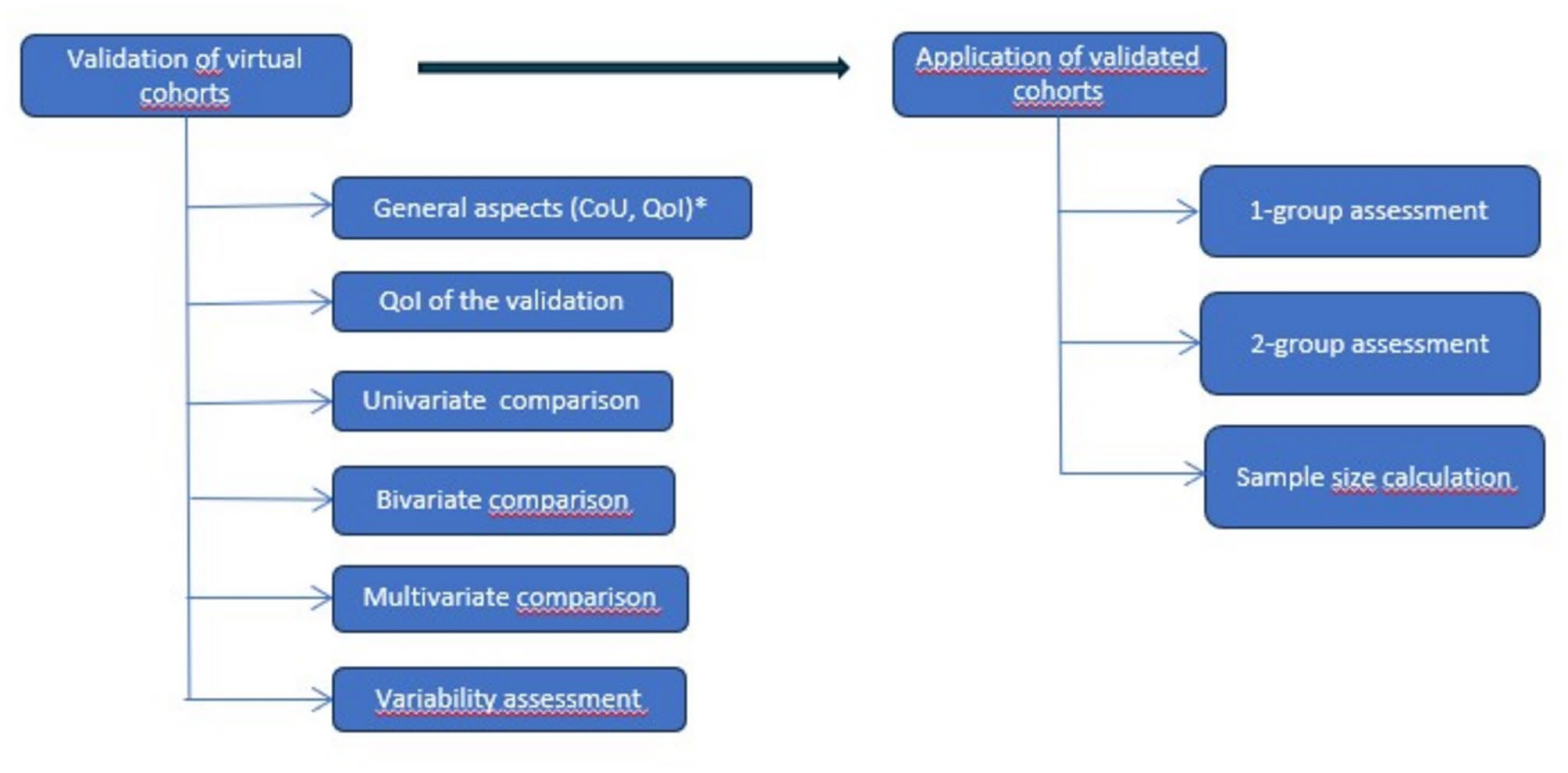
Functionality of R-statistical environment (CoU = context of use, QoI = Question of Interest).

The resulting interactive web application with R was developed in the R programming language, based on the Shiny package, and is openly available as version 0.1.0 (https://github.com/ecrin-github/SIMCor). It is intended to support proof-of-validation for virtual cohorts and computer-based simulations. It offers a series of standard analytic techniques that can be applied to compare virtual cohorts with real datasets, thus supporting the process of validation of a virtual cohort (one-, two- and multivariate comparisons). Furthermore, it provides different options to apply validated virtual cohorts in in-silico trials. This covers a one-group assessment (no control) and two-group comparisons together with options on statistical design and sample size calculations (Fig. 1). The tool is generic and menu driven and it provides user guidance and help. Additional information on software development is documented in S6 Development history of the application and R-packages integrated in the R-statistical environment.

### General aspects

Validation and application of virtual cohorts are related to the Context of Use (CoU) and the Question of Interest (QoI). Definitions for these terms are taken from the FDA Guidance on “Assessing the credibility of computational modelling and simulation in medical device submissions”^20^. For models used in in-silico device testing or in in-silico trials, the CoU should describe how the model will be used in a simulation study to address the QoI. The QoI defines the specific and concrete question related to the CoU. As such CoU and QoI are prerequisites for any kind of validation or application activity directed at virtual cohorts or in-silico trials. CoU and QoI are essential elements for the scope and role of the computational model and the specific question addressed. Therefore, documentation of this information is necessary for the application of the R-statistical environment.

### Implemented functionality

#### Validation of virtual cohorts

To apply the module “validation of virtual cohorts”, as a first step, the **CoU,** and the **QoI** must be specified as free text and submitted (by clicking the boxes).

**Fig. 2:**
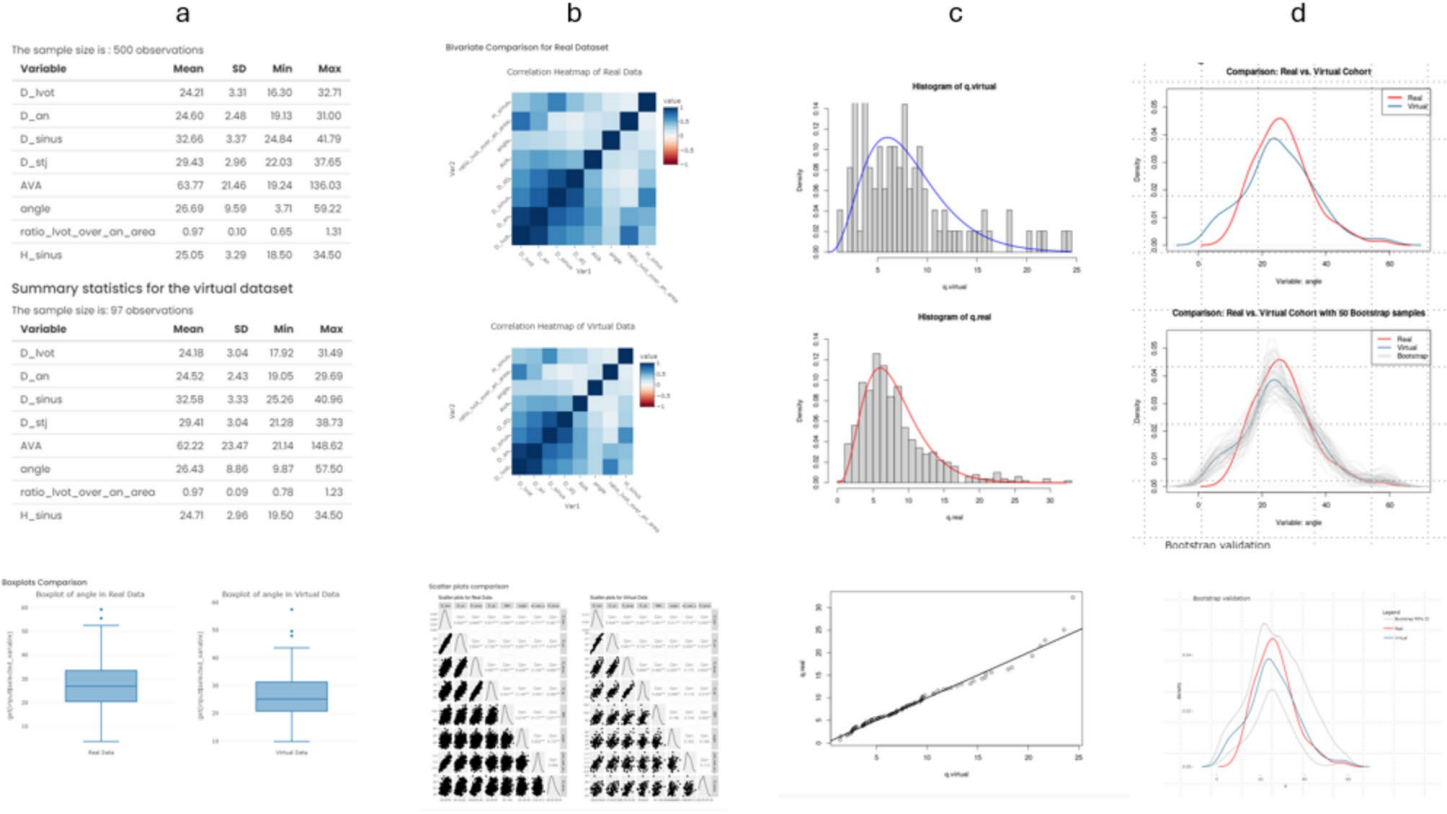
Screenshots from the module “Validation of a virtual cohort” (**a**) Univariate comparison, (**b**) Bivariate comparison, (**c**) Multivariate comparison, (**d**): Variability assessment.

The next step deals with the upload of datasets for validation including both the virtual dataset and the real dataset to be compared to. For the import, any accessible computer can be browsed, and a specific file selected. Virtual and real datasets must be uploaded as a CSV file with the same variable structure. After uploading, the following analysis can be performed (see: Fig. 2 with screenshots and Table 1):

**Table 1:** Analytical techniques implemented in the R-statistical framework.

Module	Section	Algortihms
Validation of virtual cohorts	Univariate	Mean value, standard deviation, minimum and maximum Scatter plots of combinations of variables
	Bivariate	Spearman correlation coefficients between all features (separately for real and virtual data) Correlation heatmaps and plots
	Multivariate	Quantile–Quantile plot between the synthetic and the real data after multivariate standardization of each data sets
	Variability assessment	Bootstrap technique with the real data taken as a reference distribution and the bootstrap samples generated from the virtual dataset 95% confidence bounds for the density function Results graphically displayed as density functions
Application of validated cohorts	One group assessment	Frequency and a chi-square test for a discrete variable, a boxplot for a continuous variable, and a Kaplan–Meier curve for a time-to-event variable
	Two group assment	Frequencies for discrete variable, boxplots for continuous variable and Kaplan–Meier curves for time-to-event variable Chi-square test for a discrete variable, a t-test for continuous variable and a Kaplan–Meier test for time-to-event variable
Sample size calculation		Two-group comparison and continuous outcome variable with a common standard deviation taking uncertainty under consideration

#### Univariate comparison

From the imported data sets all, several or one specific variable is selected and separately for the virtual and real datasets descriptive statistics are calculated and presented:

- Mean value, standard deviation, minimum and maximum
- Scatter plots of combinations of variables

The results are presented as tables with variables as rows and separately for virtual and real data. In addition, the results are shown as box plots (see Fig. 2a).

#### Bivariate comparison

Here, separately for the real and virtual dataset, bivariate correlations between the variables are calculated. The idea is to compare the correlations within the two cohorts, to not only assess whether parameter distributions, but also their relations are adequately mimicked by the virtual cohort.

The results are graphically displayed as so-called heatmaps. Correlation heatmaps are a type of plot that visualise the strength of relationships between numerical variables. Correlation plots are used to understand which variables are related to each other and illustrate the strength of this relationship (see Fig. 2b).

#### Multivariate comparison

To evaluate the compatibility of the virtual cohort with the real data, a multivariate comparison between the n-dimensional distributions of the features of both cohorts can be performed in the R-statistical environment (see Fig. 2c). The following test is used:

- Quantile–Quantile plot between the synthetic and the real data after multivariate standardisation of each data set. Multivariate standardisation is performed by (1) subtracting the vector of means to each vector data point and (2) scaling by using the inverse of the variance covariance matrix. The resulting standardised quantity is a quadratic form that under the assumption of multivariate normality, it follows a chi-squared distribution with degree of freedom equal to the number of variables minus one. The histogram of these quadratic forms are displayed for the real and virtual data. The methodology is explained in the supplementary material S5.

#### Variability assesment

In this validation approach, the results from the model (virtual dataset) and the experiments (real dataset) are plotted together using a nonparametric density function for a variable of interest. The uncertainties associated with the model (arising from input uncertainties and numerical uncertainties) and the experiment (stemming from measurement system uncertainty and specimen-to-specimen variability) are represented in the two distributions. Any discrepancies between the two curves are thus interpreted as uncertainty in the model that generated the virtual data.

The uncertainty in the model is assessed by a bootstrap technique. The real data is taken as a reference distribution, and bootstrap samples are generated from the virtual dataset. For each bootstrap sample, a nonparametric density function is calculated, and 95% confidence bounds for the density function are calculated. If the real data is not covered by the 95% confidence bounds created from the virtual dataset, then a deviation of the model has been detected.

In addition, a bootstrap *p*-value can be calculated from the number of times that the density of the real data is out of the 95% confidence bounds of the bootstrap analysis. The results are graphically displayed as density functions (see Fig. 2d). The methodology is described in supplementary material S5.

#### Application of validated cohorts

**Fig. 3:**
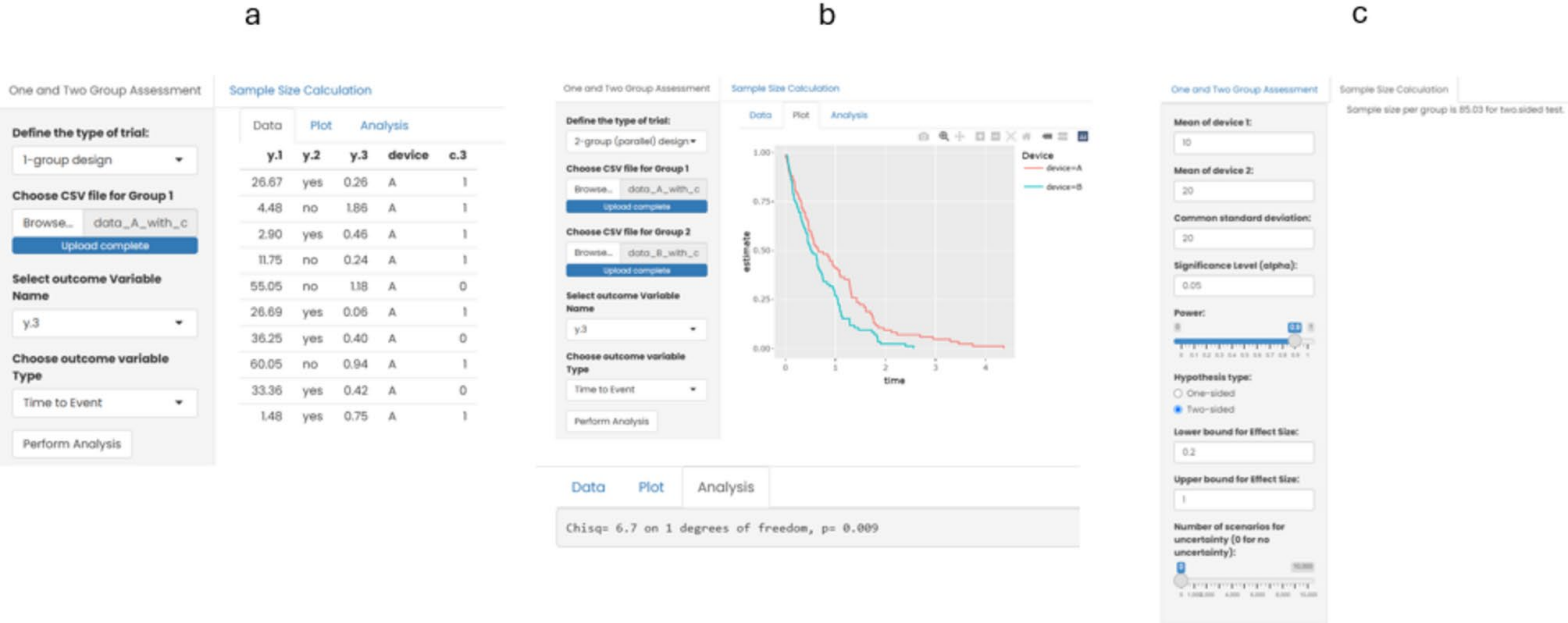
Screenshots from module “Application of validated cohorts” (**a**: 1-group design, **b**: 2-group design, **c**: Sample size estimation).

The following analytical techniques are implemented in the R-statistical environment (see Fig. 3 with screenshots and Table 1):

#### One-group design

For the 1-group design (i.e. only one validated cohort and no control), the dataset for the analysis should be imported as, currently, CSV files. For the import, any accessible computer can be browsed, and a specific file selected. Then the variable of interest should be specified by selecting from the list of all variables of the dataset imported. In the next step, the type of variable needs to be specified (discrete, continuous, time to event) (see: Fig. 3a).

If “perform analysis” is clicked, then there are 3 options available:

- Data (default)
- Plot

Under “Data” the individual records belonging to the dataset can be browsed.

“Plot” provides a figure reporting frequency and a chi-square test for a discrete variable, a boxplot for a continuous variable, and a Kaplan–Meier curve for a time-to-event variable.

#### Two-group design

For the 2-group design, the two datasets for the analysis should be imported as, currently, CSV files. For the import, any accessible computer can be browsed, and two datasets should be selected sequentially. The structure of the two datasets should be the same. Again, and like the 1-group design, the variable of interest should be specified by selecting it from the list of all variables of the datasets imported. In the next step the type of variable needs to be specified (discrete, continuous, time-to-event) (see Fig. 3b).

If “perform analysis” is clicked, three options are available:

- Data (default)
- Plot
- Analysis

Under “data” (default option), the individual datasets can be browsed.

For a discrete variable, “plot” provides a figure reporting frequencies for the two datasets. For a continuous variable, boxplots of the two datasets are presented. For a time-to-event variable, the two Kaplan–Meier curves are shown in one figure.

The function “analysis” covers a chi-square test for a discrete variable, comparing the two datasets. For a continuous variable, a t-test is presented and for a time-to-event variable a Kaplan-Meier test.

#### Sample size estimation

The module on sample size calculation is currently restricted to a two-group comparison and a continuous outcome variable with a common standard deviation between the groups. For application of this function, the mean for group 1, the mean for group 2, the common standard deviation, the significance level, the power, and the hypothesis type (one- or two-sided) need to be entered.

For taking uncertainty in the estimation of effect size into consideration, a lower bound (minimum 0) and a higher bound (maximum 1) can be defined. Finally, the number of scenarios can be specified with the slider. If all information is entered, a summarising statistic for the calculated sample size with minimum, median, mean and maximum is given. Probabilities for achieving a power less than 90% for different sample sizes are calculated and presented (see Fig. 3c).

For any individual statistical algorithm applied in an analysis within the R-statistical environment, a report in pdf-format is available.

### Testing and validation

Testing of the R-statistical environment covered inspection of the Shiny code, module testing, and integration testing of the full application. A senior statistician was involved in this process (PEV).

In addition, the tool application was validated against concrete examples:

#### Validation of virtual cohorts

Verstraeten et al. developed a virtual cohort generator that creates anatomically plausible, synthetic geometries of stenosed aortic valve geometries for use in virtual TAVI trials^21^. This generator was constructed utilising an approach that combines non-parametric statistical shape modelling with sampling from various distributions. It was able to produce 500 synthetic aortic valve stenosis geometries, which were then validated by comparison with a real-life cohort of 97 patients, confirming the validity of the virtual cohort. The R-Statistical environment resulted in reproduction of the original results and allowed further extension of the study’s analysis. The data used in this study, including the examples, are publicly accessible on 4TU.ResearchData^22^ and on ECRIN’s GitHub repository^23^, specifically under the filenames “shapeFeatures_real.csv” and “shapeFeatures_synthetic.csv”.

#### Application of validated cohorts

The data used for validation was generated through simulations for a study comparing two devices, labelled as Device A and Device B, across three types of outcome variables: continuous, discrete, and time-to-event. The data were independently analysed with separate R-scripts and the results were compared with those from the R-statistical environment.

The datasets needed for these two examples are included in ECRIN’s Github (data_A_with_censor.csv, data_B_with_censor.csv)^23^.

### Implementation as open source and in the VRE

The R-statistical environment is fully open-source, and all necessary information for its implementation, including the source code, can be found in ECRIN’s GitHub repository^23^**.** It is registered in ZENODO (https://zenodo.org/records/14718597). In addition, it can be deployed on-premises for local use or accessed as a Software-as-a-Service (SaaS) via the Virtual Research Environment (VRE) developed under the EU-funded SIMCor project. Its modules are accessible at:

- Validation module: https://simcor.unitbv.ro/shiny/validation-module/
- Application of validated cohorts’ module: https://simcor.unitbv.ro/shiny/application-module/

The SIMCor VRE represents the computational platform designed and implemented during the project for the design, conduction, and result analysis of in-silico clinical trials, with a focus on modelling and simulation to assess the conformity, safety, and efficacy of cardiovascular devices^24^. In its current online version, it covers the VRE Drive, a data repository and interoperability environment for the exchange of data among the various VRE components, the virtual cohort generator for the generation of a new population of virtual patients, based on a set of characteristics provided as input parameters, a module for virtual TAVI device Implantation and the R-statistical environment.

The VRE is also referenced in a SOP about virtual cohorts generation and validation^25^.

### Application of the R-statistical environment in SIMCor

In the SIMCor-project three concrete in-silico trials were performed:

- TAVI-1: Effects of convergent and divergent Left Ventricular Outflow Tract (LVOT) shapes on paravalvular leakage (PVL) after virtual TAVI
- TAVI-3: Effects of calcification on PVL after virtual TAVI
- PAPS-1: Effects of pulmonary artery side branches on haemodynamics and fixation safety of PAPS, to assess whether detailed assessment of the landing site during device implantation is necessary.

The procedure started with the formulation of application scenarios. Thereafter the study protocol was developed, including a detailed description of the datasets. Then, the virtual cohorts for the in-silico trials were generated and finally, the data analysis was performed.

Due to time reasons, the analysis in the three in-silico trials could not be performed in the R-statistical environment but was done separately with R studio. It was, however, assessed whether the different analysis procedures applied in the in-silico trials could have been performed within the R-statistical environment. The results of this comparison are summarised in Table 2:

**Table 2:** Statistical analysis of in-silico trials in SIMCor.

Statistical analysis	In-silico trials		
	TAVI-1	TAVI-3	PAPS-1
1-group	**Bivariate correlations** Linear mixed-effect models	**Univariate descriptive analysis** **Bivariate correlations** Multivariate linear regression Logistic regression	**Univariate descriptive analysis** **Bivariate correlations** Analysis of variance Linear mixed-effects model
2-group	–	–	–
Sample size	–	**Sample size calculation**	–

Bold = covered by the R-statistical environment , normal = not yet covered by the R-statistical environment).

It turned out that the basic techniques could have been applied in the R-statistical environment, however, more sophisticated techniques (e.g., linear mixed-effects model) are still missing in the application.

## Discussion

In biomedical, pharmaceutical and toxicology research, the safety and efficacy of biomedical products is ultimately tested on humans via clinical trials after prior laboratory testing in-vitro and/or in-vivo on animals. The complete development chain of a new biomedical product and its introduction to the market is very long and expensive. Alternative methodologies to reduce the animal and human testing are needed to address the safety and efficacy issues of clinical human trials, the ethical issues and the imperfection of predictions issued from laboratory and animal studies when applied to humans^26^. Regulatory bodies, such as the FDA, are more and more realising, that computational (in silico) modelling and simulation (M&S) are powerful tools that complement traditional methods for gathering evidence–including bench-top (in vitro) testing, and animal or clinical (in vivo) studies–about products regulated by FDA or for developing FDA policy^20,27,28^. In -silico medicine may significantly speed up the design process and reduce costs in medical device and medicinal product R&D by enabling virtual prototyping and testing through computational models^29^. As a consequence, standards, frameworks and guidelines have been developed characterising and promoting in-silico trial technologies (e.g.,^1,30–34^).

Similarly, increasing uptake of in-silico trials will require to ensure replicability and reproducibility of findings and model outcomes and thus their credibility. Even though the ongoing “replication crisis” is not only affecting computational biomedical research, this field is particularly sensitive due to the use of complex in-silico models and data, usually used for model parameterisation, which are often not freely available due to intellectual property or data privacy-related issues^35,36^. To address this issue robust tools for planning and evaluation of in-silico trials, which facilitate systematic, complete and automatic documentation of all individual steps performed are considered to be strongly beneficial. This aspect was specifically targeted in our study for the R-statistical environment by adequate integration of a report function, fostering systematic and complete documentation of all individual steps performed in the planning and analysis. This can be considered as a significant contribution to reduce the replicability crisis in the field of computational modelling and simulation, admitting that this is only a small part of the full development and application chain for virtual cohort generators.

A general issue to be solved for the area of virtual cohorts and in-silico trials is to provide standardised and harmonised metadata, characterising computational models but also statistical algorithms applied in the analysis of data related to it. That there are still issues with reproducibility/replicability has been demonstrated in several publications (e.g.^37^). The implementation of reproducible research for *in-silico* analyses requires extensive metadata to describe both scientific concepts and the underlying computing environment and metadata provide context and provenance to raw data and methods and are essential to both discovery and validation^38,39^. Metadata standards should be applied across the full „analytical stack “ consisting of input data, tools, reports, pipelines and publications^36^. An adequate metadata model serves to support discoverability of a virtual cohort by describing the underlying model and the creation of the virtual cohort (FAIR data^40^). In addition, metadata on the data upload should be included. Similar information could be included for the data usage and analysis. Glossary of terms for computer modelling & simulation, such as the second release of the Avicenna glossary are a first step in the right direction but not sufficient^41^. Different ontologies for modelling and simulation are available but difficult to apply and far from being standardised^42^. Metadata for statistical tools are data (information) used to describe statistical objects^43^. Statistical metadata are best understood as structured information. One of the next steps should be to extend the R-statistical environment with standardised metadata. Another area of relevance would be to link the R-statistical environment to the CDISC (Clinical Data Interchange Standards Consortium)-standard for clinical trials. First attempts in this direction have been taken in the SIMCor project but much more work is needed^44^.

To avoid redundancy and unnecessary work, the development of a new tool should only be triggered, if the functionality needed is not covered by already existing tools. For that reason, we performed a survey, trying to assess the status of existing computer tools for support of analysis of virtual cohorts and in-silico trials. Only R packages, known for their widespread use, that were listed in CRAN were included in the search as these packages are validated by the R foundation^45^. In total 8  applications could be identified, relevant for computational modelling. Closer consideration of the tools revealed that the target of these tools was limited to specific aspects, such as simulation of specific trial types (e.g., platform trials, pharmacokinetic-pharmacodynamic studies, adaptive trials) or application of Bayesian techniques (supplementary material S1). So, some of the tools cover aspects relevant for supporting the validation of virtual cohorts or applying virtual cohorts in in-silico trials but none of the tools seemed to be generic enough to cover the functionality needed in the SIMCor project.

The choice to use the programming language R and the Shiny package to develop the web application was influenced by their popularity, open-source nature, and the extensive international community that supports them. One might question why other options were not considered for developing the tool and whether this decision significantly limits its applicability. An alternative approach involves using the Python programming language, which is versatile and commonly utilized for data science projects, such as building websites, automating tasks, and processing images and text statistically. Python is a functional programming language similar to R and integrating both languages is straightforward. For instance, the reticulate package offers an R interface to Python modules, classes, and functions^46^. Conversely, it is also possible to run R code from Python using the rpy2 package^47^. Additionally, RStudio allows users to work with both languages within an integrated development environment. Therefore, choosing R as the statistical tool for this project should not be viewed as a limitation on its usage or applicability.

An R-statistical environment alone is certainly of benefit for potential users, but it realistically only covers part of the pipeline related to the development and application of virtual cohorts as well as in-silico trials. Strengths lie in the possibility to upload any dataset from any domain (currently only CSV) and to apply statistical algorithms, which are of value and used for the validation of virtual cohorts and the application of validated cohorts in in-silico trials. It would be, however, a major step forward, if the statistical techniques implemented in the R-statistical environment could be coupled with computational models for generating virtual cohorts (so-called virtual cohort generators). This would allow it to support the workflow from generating virtual cohorts with computational models to analysis of the generated data in one environment. This approach was followed in the SIMCor-project by integrating virtual cohort generators with the R-statistical environment in a VRE^24^. The VRE still has some limitations, so the use is currently restricted to specific users and the full pipeline of computational modelling is not integrated yet with a need to connect local environments for complex simulations. Nevertheless, the VRE is a necessary and promising step towards an open and powerful computational environment for developing and applying virtual cohorts.

The R-statistical environment developed in SIMCor is a first and promising step, but it needs considerable further improvement. The availability of statistical algorithms for analysis of virtual cohorts and of tools to plan in-silico trials is still limited. In the three in-silico trials of the SIMCor project it turned out that much more statistical techniques need to be covered by the tool to be useful. As an example, linear mixed-effects models had to be applied here in two of the three in-silico trials performed in SIMCor (TAVI-1, PAPS-1), which was done outside the R-statistical environment. Many more techniques can be named that could be of benefit for potential users when dealing with virtual cohorts and in-silico trials (e.g. multivariate logistic regression, sample size estimation for in-silico trials based on odds ratio).

Finally, the R-statistical environment was developed within the SIMCor project and thus any experiences with the application so far are limited to the domain investigated and the project partners. The next step should be to gain more experience by applying the tool by other researchers, in other domains and for other questions. With the code and supporting information (implementation and user guide) openly available in GitHub and the underlying statistical techniques fully described in the model description, this should be feasible. A concrete next step is to make the software available under CRAN. CRAN is a network of ftp and web servers around the world that store identical, up-to-date, versions of code and documentation for R. Co-author PEV has major experience with CRAN and has implemented several packages under CRAN (Jarbes, bamdit). Furthermore, the entirety of the SIMCor Virtual Research Environment, including its modules, such as the R-statistical environment have been included in the EDITH catalogue. This project is establishing the foundation of the European Virtual Human Twin infrastructure, which will be implemented subsequently. It is envisaged to include the R-statistical environment within this framework together with its application examples. On a shorter time frame, the application scenarios described in this work will be individually published, demonstrating the methods and findings of each respective in-silico trial. They will directly refer to the use of the R-statistical environment for the design and evaluation of the in-silico trial and therefore provide comprehensive application examples for the tool.

## Conclusions

In this project, an open, generic, and menu-driven web application has been developed in Shiny to support the validation and application of virtual cohorts in specific use cases related to cardiological medical devices. The tool is applicable to other use cases, research environments, and scientific domains for the development or regulatory evaluation of a medicinal product, device, or intervention.

## Electronic supplementary material

Below is the link to the electronic supplementary material.

Supplementary Material 1

## Data Availability

Code and examples are available unde**r** ECRIN GitHub: SIMCor. https://github.com/ecrin-github/SIMCor. and ZENODO: https://zenodo.org/records/14718597. A detailed description of the R-statistical environment (including survey, user stories) is available under https://drive.google.com/drive/folders/1_cRFCkLn1mYbUjy_yrOxOwdtgmkNQXCM (SIMCor google drive). This file will be published in ZENODO in case the manuscript is accepted for publication. The example for “validation of a virtual cohort” (Verstraeten et al.) is available under https://data.4tu.nl/datasets/3f6a3788-96e6-4b81-b37b-f07eeec85965
