# Supplementary material for: An open source statistical web application for validation and analysis of virtual cohorts
Source: Sci Rep. 2025 May 6;15:15744. doi: 10.1038/s41598-025-99720-3 (PMC12056029; doi:10.1038/s41598-025-99720-3)
Supplement: Supplementary file 1 — Supplementary Material 1 [file 41598_2025_99720_MOESM1_ESM.pdf]

Supplementary material for

An open source statistical web application for validation and analysis of virtual cohorts

Results from the SIMCor project

Authors

Christian Ohmann*
European Clinical Research Infrastructures Network (ECRIN), Kaiserswerther Strasse 70, 40477, Düsseldorf, Germany

Takoua Khorchani
European Clinical Research Infrastructure Network (ECRIN), 75014, Paris, France

Alexandru Cracanel
Automation and Information Technology, Transilvania University of
Brasov, Mihai Viteazu nr. 5, Brasov, 5000174, Romania

Jan Brüning
Institut für kardiovaskuläre Computer-assistierte Medizin, Charité, Universitätsmedizin, Berlin, Germany

Pablo Emilio Verde
Coordination Centre for Clinical Trials, Heinrich Heine University Düsseldorf, 40225, Düsseldorf, Nordrhein-Westfalen, Germany

-

Corresponding author*

Prof. Dr. Christian Ohmann, European Clinical Research Infrastructure
Network (ECRIN), Kaiserswerther Strasse 70, 40477, Düsseldorf, Germany,
email: christianohmann@outlook.de

S1: Survey about existing R-tools

R-packages for supporting statistical analysis of virtual cohorts

Only R packages that are listed in CRAN are included. These packages are validated by the R foundation. Packages at GitHub repository that also are at CRAN are developing versions. Packages only at GitHub are not considered validated and therefore have not been included.

Name	Short description	URL	Open source	Licence	Target
adaptr	Package that simulates adaptive (multi-arm, multi-stage) clinical trials using adaptive stopping, adaptive arm dropping, and/or adaptive randomisation.	https://cran.r-project.org/web/packages/adaptr/index.html https://joss.theoj.org/papers/10.21105/joss.04284	Yes (CRAN, github)	GPL (≥ 3)	Simulation of adaptive clinical trials
PopED	The Population Equivalency via Simulation (PopED) package	https://cran.r-project.org/web/packages/PopED/index.html	Yes (CRAN, github)	LGPL (≥ 3)	Simulation of pharmaco -kinetic-pharma-

	facilitates the design and analysis of pharmacokinetic - pharmacodynamic (PK-PD) studies. It allows for population modeling, trial simulation, and evaluation of dose-response relationships to optimize drug dosing in clinical trials using virtual populations.				codynamic studies
Mediana	Mediana is an R package which provides a general framework for clinical trial simulations based on the Clinical	https://cran.r-project.org/web/packages/Mediana/vignettes/mediana.html	Yes (CRAN and github (development version))	GPL-2	Clinical trial simulations supporting a broad class of trial models.

	Scenario Evaluation approach. The package supports a broad class of data models (including clinical trials with continuous, binary, survival-type and count-type endpoints as well as multivariate outcomes that are based on combinations of different endpoints), analysis strategies and commonly used evaluation criteria.				

cats	Cohort plAtform Trial Simulation whereby every cohort consists of two arms, control and experimental treatment. Endpoints are co-primary binary endpoints and decisions are made using either Bayesian or frequentist decision rules. Realistic trial trajectories are simulated, and the operating characteristics of the designs are calculated.	https://cran.r-project.org/web/packages/cats/index.html	Yes (CRAN, github)	MIT + file LICENSE	Simulation of cohort platform trials investigating combination treatments
CRAN task view	Clinical Trial Design, Monitoring, and Analysis	https://cran.r-project.org/web/views/ClinicalTrials.html<br>*Search in "CRAN task view Clinical Trial Design, Monitoring, and Analysis" with the key word "simulation" revealed 8 R-packages, of which the 2 relevant for this work were already included in the survey.*	Yes (CRAN, github)	unknown	Not about simulation of trials

NCC	An R-package for analysis and simulation of platform trials with non-concurrent controls	https://www.sciencedirect.com/science/article/pii/S2352711023001334 https://pavlakrotka.github.io/NCC/index.html https://cloud.r-project.org/web/packages/NCC/index.html	Yes (CRAN, github)	MIT + file LICENSE	Analysis and simulation of platform trials with non-concurrent controls
BACCT	This package implements the Bayesian Augmented Control (BAC, a.k.a. Bayesian historical data borrowing) method under clinical trial setting by calling ‘Just Another Gibbs Sampler’ (‘JAGS’) software.	https://cran.r-project.org/web/packages/BACCT/index.html	Yes (CRAN)	GPL (≥ 3)	Simulation of type-I error/ power, or probability of correct go/no-go decision at interim look
bayesCT	This package performs simulation and analysis of	https://cran.r-project.org/web/packages/bayesCT/vignettes/bayesCT.html	Yes (CRAN, github)	unknown	Simulation and analysis of Bayesian

	Bayesian adaptive clinical trials for binomial, Gaussian, and time-to-event data types, incorporates historical data and allows early stopping for futility or early success.				adaptive clinical trials
BayesCTDesign	This package provides a set of functions to help clinical trial researchers calculate power and sample size for two-arm Bayesian randomized clinical trials that do or do not incorporate historical control data.	https://cran.r-project.org/web/packages/BayesCTDesign/index.html	Yes, (CRAN, github)	GPL-3	Power and sample size for two-arm Bayesian randomized clinical trials

S2: Minutes from Workshop

SIMCor

In-Silico testing and validation of Cardiovascular IMplantable devices

Call: H2020-SC1-DTH-2018-2020 (*Digital transformation in Health and Care*)

Topic: SC1-DTH-06-2020 (*Accelerating the uptake of computer simulations for testing medicines and medical devices*)

e-workshop

Statistical environment for in-silico trials

# - Meeting notes -

Participants

Maria Panagiotopoulou (ECRIN, SIMCor WP10), Pablo Verde (University of Düsseldorf), Christian Ohmann (ECRIN, SIMCor WP10), Jan Brüning (Charité, SIMCor, WP1,5,6,9), Anna Rizzo (Lynkeus, SIMCor WP1,2), Giorgio Davico (University of Bologna, In Silico World), Akanksha Sachan (ETH Zürich), Raphaël Porcher (Université de Paris), Jacob Sturdy (Norwegian University of Science and Technology), Antonino Amedeo La Mattina (University of Bologna, In Silico World), Sarah Zohar (Inserm-Inria), Christian Niklas (University Hospital Heidelberg), Michele Barbier (Inria, SimCardioTest), Ilse Van Herck (Simula, SimCardioTest), Irene Balelli (Inria, SimCardioTest), Marta Saiz Vivó (University Pompeu Fabra, SimCardioTest), Enrico Mastrostefanno (IAC-CNR), Leonid Goubergrits (Charité, SIMCor), Georgia Karanasiou (FORTH, In Silico World), Alexandru Cracanel (UTBV, SIMCor WP3), Giuseppe Alessandro Parasiliti Palumbo (University of Catania, STriTuVaD), Goran Stanic (University of Bologna, In Silico World), Wouter Huberts (Eindhoven University of Technology, SIMCor/In Silico World), Bernard Staumont (University of Liège/VPHi, In Silico World/SIMCor/SimCardioTest), Nina Krüger (Charité, SIMCor WP6), Jacques Demotes (ECRIN, SIMCor WP10), Charles Ledoux (ETH Zürich, InSilicoWorld), Roberta De Michele (University of Bologna, InSilicoWorld), Raphaëlle Lesage (VPHi, SIMCor/In Silico World/SimCardioTest), Axel Loewe (Karlsruhe Institute of Technology, MICROCARD), Paola Stolfi (IAC-CNR), Levente Sandor (Budapest University of Technology and Economics, In Silico World), Benjamin Csippa (Budapest University of Technology and Economics, In Silico World), Elena Crispino (University of Catania), György Paál (Budapest University of Technology and Economics, In Silico World), Nerea Arrarte Terreros (Amsterdam University Medical Center, INSIST), Eleni Kolokotroni (ICCS, NTUA), Cristina Curreli (University of Bologna, In Silico World), Yogesh Deepak Bansod (ETH Zürich, In Silico World), Siegfried Eisenberg (IHS, SIMCor WP10), Claudio Capelli (University College London, SIMCor WP5/ENRICHMENT), Nils Götzen (4RealSim, SimInSitu/PCoCo), Lucian Itu (UTBV, SIMCor WP3), Liesbet Geris (VPHi), Mathias Peirlinck (TU Delft), Enrique Morales Orcajo (Ambu Innovation), Maria Cristina Jori (Mediolanum Cardio Research), Marian Bubak (sano), Bruno Mario Cesana (University of Milan), Sergei Gorianin (ECRIN, SIMCor WP10), Burç Aydin (ECRIN, SIMCor WP10), Steve Canham (ECRIN)

Welcome (Maria Panagiotopoulou, ECRIN)

The SIMCor project is a 3-year H2020 Research and Innovation Action aiming at developing a computational platform for in silico development, validation and regulatory approval of cardiovascular implantable devices. The SIMCor consortium consists of 12 partners from 8 countries: Charité which is coordinating the project, Lynkeus for the project management and communication, Biotronik, ECRIN, IHS, IIB, Philips, Eindhoven University of Technology, Graz University of Technology, Universitatea Transilvania Din Brașov, University College London and VPHi. The workshop is organised in the frame of WP10 “Quantification of healthcare, industry and socioeconomic effects”.

Introduction to in-silico trials (Christian Ohmann, ECRIN)

“In-silico” is a term used in the context of computer modelling and simulation. It is in contrast to the use of “in vitro” meaning on the bench, “ex vivo” outside the body and “in vivo” inside the body. The in-silico trial refers to the use of individualized computer simulations in a cohort of patients during the development or regulatory evaluation of a medicinal product, medical device or medical intervention. There are already a series of guidelines available which describe how these trials should be performed: Guidance from the FDA, the EMA, documents from the American Society of Medical Engineers, position papers from the Avicenna Alliance and documents from the Agency for Healthcare Research and Quality. Currently, several EU projects work on the topic: SIMCor, In silico World, SimInSitu, SimCardioTest, INSIST and from the USA: FDA: Model-Informed Drug Development Pilot Program (MIDD).

In SIMCor, we looked at what is available that could be used for performing in-silico trials out of the box and we found that the existing tools & services do not perfectly fulfil our requirements. There are tools like the InSilico trial platform, the QSP (quantitative systems pharmacology toolbox), the Universal Immune System Simulator (UISS), Simulo, Highly Efficient Clinical Trials Simulator (HECT).

Thereafter some examples were presented, where an in-silico trial could present benefit. The first example is a trial about a drug for endocrine disease. Initially the investigators looked at reducing an endpoint from time zero to time six weeks. They wanted to show a significant decrease at six weeks. However, the predicted power was less than 70%. If instead 12 weeks are considered for the clinical trial, the power increases up to 80%. This is based on the simulations done. If you exclude patients with mild disease, you get a stronger effect and again the power increases.

The second example of an in-silico trial was related to Alzheimer disease. It's a 78 weeks parallel design, drug vs placebo and the endpoint is ADA score. The drug is working much better than the placebo. The question was whether another design could be followed, a delayed start, meaning that after one year both groups get the active drug. A sample size calculation was performed for the delayed design. It became clear that for the parallel design 250 patients were needed for a power of 80%, but 600 patients for a delayed start design and a power greater than 80%.

Another example is a phase III study on diabetes where the dose response of a drug on HbA1c was measured. The drug under development was compared vs a comparator, and it was estimated from the in-silico trial what dose is needed to be significantly better than a comparator. First they looked at the dose when the active drug was with a high percentage better than the comparator and then in a second step they looked at what could be the selection of the comparator. In the end the investigators showed that the simulation helped to pick up a dose and to select a suitable comparator.

In the last example, the mean effect of a drug at a selected dose should exceed 0.6. The investigators did the simulation of outcomes from 2000 subjects and they found a median mean effect of 0.74. If simulation is repeated 9 times, 3/9 times the criterion was not passed, but 9 is not a very high sample size, so 2000 replicates of 200 were performed and it was found that in 85% of the studies the mean effect was greater than 0.6 and that helped to take a decision.

In providing the framework for in-silico trials, the methodology by Bodner & Kaul (2021) is proposed. During the workshop the main steps of this approach were summarised. This methodology starts with the model development and when the model is built, the validation and verification as well as uncertainty quantification are performed. Thereafter, clinical trial planning is done. Next, you need a human cohort as well as a virtual cohort. Finally, model validation is performed using the human data. If the model is adequate, a decision is taken and the model can be used for prediction. A virtual cohort can be produced and can then be used for application in the in-silico trial. If the model is not adequate, a second human cohort may be needed for further processing.

A specific problem exists in the transfer between the engineering outputs from the model and the clinical endpoints/outcomes. Here a kind of transfer function is needed. Usually in the in-silico trials only engineering outputs are measured, however, in clinical trials, the clinical endpoints are of interest. It may be that there are surrogate endpoints already validated and used, but usually you have to make a link between the engineering output and the clinical endpoints. For example, in SIMCor and the TAVI use case, clinical endpoints are thrombosis, paravalvular leakage and durability and the engineering outputs are wall shear stress, oscillatory shear indices, residual times, recirculation regions and washouts.

This problem is tackled in a fictional example from Bodner & Kaul via a cumulative density function plot of the results of a preclinical validation. In this example cadavers were used. The experimental data are within the area resulting from the model output (including variation), indicating that the model is quite good. With a boxplot of engineering output, which is electrode migration, set in relation to the clinical output, which is complication and was measured in a cohort of 150 patients, a cut-off value could be identified, allowing good discrimination between the clinical endpoint and the engineering output; in here it's 8 mm with a variation of 1 mm.

The workflow starts with a simulation model which has already been validated. In the next step clinical trial planning is performed. The information resulting from clinical trial planning is transferred to the virtual cohort generator. After generation of the virtual cohort, the data are transferred into the analysis environment. Here the clinical trial analysis is performed. This process is then repeated in a systematic variation, according to the simulation design. If all the in-silico trials have been performed, a decision can be taken on how to proceed.

R is a software for statistics. The R statistical analysis environment planned for SIMCor in-silico trials should be able to specify the trial design and the trial data structure. In the R environment, the analysis of the in-silico trial and real trials (if data is available) is performed. If additional data are available, extra validation of the in-silico trials against real data can be performed and this has also been done within the R statistical environment. The results are stored and assessed and everything which has been done, the scripts for analysis as well as the results should be archived. The analysis environment should provide possibilities for systematic variation of trial design and datasets. So for clinical trial planning, what factors can be varied? For example, in- and exclusion criteria (e.g. exclude mild patients), include certain age groups).

The analysis of the in-silico trials covers the estimates of efficacy, the estimates of safety and also can be used to investigate the explanatory power of the in-silico trial. This process is repeated or systematically varied with a new clinical trial planning. Then the new protocol is transferred to virtual cohort generator, the in-silico trials data generated according to a new clinical trial protocol are then transferred to the analysis environment and again this is analyzed and this process is repeated many times according to a systematic variation plan. If real data are available which have not been used for the development and the initial validation of the in-silico model, they can be used to validate in-silico trials against these real clinical trials and here specific statistical techniques are needed for comparison.

In SIMCor there are 3 different components: the virtual cohort generator, the R analysis environment and the Virtual Research Environment (VRE), which can be seen as an archive, where everything is stored and made available to researchers. This means that the archiving must consider those parts of the whole procedure that are needed to repeat the analysis. This is something that needs further clarification.

How to provide an R-statistical environment for in-silico trials (Pablo Verde, Coordination Centre for Clinical Trials, University of Düsseldorf)

R is a free software for any kind of data analysis. R is open source, so you can see the code and modify it or share code in collaboration projects. R is a dialect of the S system. This system has evolved over a period of 20 years. It was a research project in the 70s and 80s at the Bell Laboratories in USA and the aim was to develop a statistical working environment, where new ideas can be very simply implemented and used for further analysis. The work in R from J. Chambers was awarded by the ACM. Previous awards of ACM were the UNIX system, the World Wide Web, the TCP/IP and the Postscript language.

The R system was originally written by Ross Ihaka and Robert Gentleman at the University of Auckland in 1994. In 1997 a core team of 9 members started to develop R extensively. Now the team has 20 people and the first version of R was issued on 29 February 2000. R is updated about three times in a year and it has been very popular over the last 10 years.

There are reasons for R's success; one was the early decision of releasing R with a GNU General Public License which makes R basically publically available. The R project attracted very talented people and there was a lot of synergy so at the beginning of 2000s R started developing in a quick and robust manner. There's another strategic decision which was how the R packages were invented. The R package is sort of a piece of software which is used to extend the basic functionality of R and these R packages have a very well-defined structure where you have documentation of all the functions. Now there are about 18,000 packages.

For the technical people, R follows a functional programming style so computations are organizing their own functions, which encapsulate the specifics and meaningful computational task. The functions link with methods. The functions basically are applied to specific objects and those objects are organized around classes of objects. The functions encapsulate actions within the software, the classes encapsulate the nature of the object. So, the objects could be basically data objects or could be also functions, a class which can perform some specific tasks.

A clinical trial is a complex data science project where you have to deal with hundreds of tables; they have to be organized, properly prepared for statistical analysis and then the statistical analysis has to be performed in a way that results are replicable.

The idea of the dynamic reporting is that in a single document you have your code for doing your statistical analysis and text which is called narratives. Once you can compile the whole R code and all of your text then you can generate a document which is basically replicable. At the beginning, dynamic reporting was difficult to use. But during the last years there was more development in the direction of using R-markdown. The R-markdown document is basically a plain text file which has the extension .Rmd. It has three main components: metadata, text and code. The metadata follows this language which is called YAML (Yet Another Markup Language). The syntax of the text follows markdown and then the R code is organized in chunks, which contain the block of R code and have to start with this symbol “’ {r} and in the text you can also report R results or statistical results. So it's sort of inline R code and if you want to use this kind of functionality you have to use ‘r.

During the workshop this was shown in a real example. The demonstration referred to an HIV comparative trial where 2 antiviral drugs where compared. 230 patients were randomized to one of the treatments and 238 were randomized to the other. Outcomes of interest were overall survival, CD4 cells measure at the baseline at 2, 6, 12 and 18 months. Also, previous opportunistic infections were part of the outcome variables measured in the trial. During the demonstration the data were analysed and it was shown how dynamic reporting looks like. The example referred to a multicenter, randomized open label community-based trial that was powered to 80% to detect particular clinically relevant effects at a statistical level of 5% two-sided.

There are some advantages of using Bayesian statistics for data analysis. From the viewpoint of a statistician this can be seen as complementary way to analyze data. The main difference in Bayesian analysis is that you have a full probability model. The Bayesian statistics have their name from the Bayesian theorem where you update the model with what you know about the models when you observe new data. This is called the posterior distribution.

The procedure has several advantages. One is that you can propagate uncertainty in a more realistic way. This is something that if you do, for instance classical statistics, or if you do simulations based on fixed number on your parameters, you are not really propagating all of the uncertainty. Bayesian statistics also allows to combine multiple sources of evidence in a coherent way. And this is performed through the prior distribution. There is another thing called posterior predictions. So posterior predictions are something that is coming more or less automatically from a Bayesian model and can be

used for a model validation. Actually, if you fit a model to data then you can compare the posterior predictions against the data that have been used for modelling and for statistical analysis. This is very useful because you can assess the limitations of the model.

The Markov Chain Monte Carlo (MCMC) has been around for many years and is a numerical way to approximate posterior distributions in very simple or very complex Bayesian models, and this is something that can be applied in any probability model. So, it doesn't matter if it's a probability model that you use to analyse the data or to generate data like in in-silico trials. There's a lot of freely available software like OpenBUGS, JAGS, Stan, Nimble fully integrated into R.

In summary, R is a very robust computational environment for statistical analysis. The dynamic reporting tools in R-Markdown provide a replicable framework for data analysis. And in-silico trials may benefit from R & R-markdown in the reporting and evaluation of results.

Roundtable discussion with participants

Question: I'm more familiar with Python and Python based analysis. Is there an equivalent that would work with Python and the Python influenced Jupyter notebooks that can include the R-markdown as well?

Pablo: There are packages for integration of Python in R. If you have a code in Python that you want to use for your particular analysis, then you can basically generate an R-markdown document where you integrate Python calculations. They can be complementary. Statisticians are more familiar with R, machine learners use more Python. What is important is to have a single document, project or template that at the end demonstrates that the results are replicable.

Question: It may be that for the in-silico trials we need specific trial designs because we might come to the point that sequential design would be better or that we say an adaptive trial would be more beneficial if we do a re-calculation of the sample size after an interim analysis. How far is R able to support these different designs?

Pablo: In the last 10 years the R project has been supported by the pharma industry. So, there are a lot of R packages that deal only with clinical trials. In the R project there is something that is called task views where packages are listed by the aim of the package. There is a complete task view on analyzing clinical trials and you can find packages for instance for sequential trials, adaptive trial, sample size calculations, classical group sequential trials etc. Also, there is a document for classical clinical trials regarding the validation of R for submitting results from a clinical trial. You can use that for in-silico trials but it might be worth exploring creating specific packages for in-silico trials in R.

Christian: Yes, and if you have developed such a script specifically for a certain type of in-silico trial and if it has been developed, tested and validated, you can use it for as many repetitions and virtual cohorts as you want.

Pablo: The other advantage is transparent documentation. You have to document in R each function that you develop and also when you submit a package to the project, this documentation will be automatically validated and your package will be rejected if you generate a function without documentation.

Christian: In SIMCor we have three major infrastructures: the virtual cohort generator, which is generating the cohorts based on the validated models, we have the Virtual Research Environment as an archive of all relevant components (e.g. models, virtual cohorts, clinical trials) and we have the R statistical environment. It has to be discussed and specified how the interaction between these 3 infrastructures should be performed. For example, data from the virtual cohort generator should be transferred to the statistical environment and then the question arises, which standards, which formats are accepted, are there restrictions? How to best organise the transfer between these 3 infrastructures?

W: Maybe what is important to discuss is where we start the statistical environment and where we start the virtual cohort generation. Because we generate virtual cohorts based on physics-based models and what we then do is actually give an intervention to that patient and then we get an outcome. I would say that the input of the statistical environment is the intervention on the virtual patient. In a normal clinical trial, we have a patient and we give that patient a drug or other intervention and then you get an effect and that effect should be the input of the statistical environment where you then do the “real” statistical analysis. But of course we need to feed back so that we can also adapt, for example in- an exclusion criteria.

Christian: The effect simulation, the simulation of the intervention is defined and implemented in the model?

Question: Yes, but the virtual patients are based upon a physiological model plus some relevant parameters and boundary conditions. So that’s the patient and at some point we put in an intervention, for example a TAVI device; at what point do we think that the clinical trial starts, is that the moment that we put virtually the device? Should that already be implemented in the statistical environment or is it after implementing the device? I think after the intervention.

Christian: Yes, it's after the intervention because otherwise we would only have a control group without treatment.

Question: Do we have to archive all the virtual cohorts, the individual data from the simulated patients which are used for the statistical analysis?

W: The way we now generate the virtual cohorts is by using surrogate models so it's very fast. We can reproduce them easily and also the models are deterministic. I would say that after the intervention that data should be archived, but not necessarily the models.

S: But like any clinical trial, you have some data storage obligation legally, which means that if you used in-silico in addition to your clinical trials in order to conclude, the legal obligation is to store everything that allows you to have an EMA or FDA approval of a drug or a medical device. And you need also to store your R version and your package version to be sure your results can be reproducible.

L: From discussions with the EMA it seems that they are interested in the clinical aspects of the in-silico trials. The accent is often put on clinical trials and how they can extend the range of what clinical data can do. So, it would be a good suggestion to have a meeting with them on this particular subject: What is it that you need and how do we need to store this and have examples that are tangible for them, preferably with a drug application or a medical device.

Christian: This is a good suggestion and since regulators can be hard to get maybe this meeting should be arranged commonly by the in-silico projects currently running.

Question: Just to clarify, are all the features you mentioned and described available in the open-source editions of R studio? Because there are also commercial editions for both the server/desktop.

Pablo: All of the tools that I presented are free; open source and not commercial. You can run the whole report from a command line in Linux so you don't need R studio neither.

Question: Going back to this issue of archiving, I think it can quickly become messy when there are lots of files, unless there is a strict naming convention around files and date stamping them etc. and also a convention about where files are stored and the rights. Is that possible within R studio? Can you impose constraints or would this have to be a separate system?

Pablo: No, you don't need a separate system.

Question: When talking about the naming conventions, would it be useful to define the parameters used in the in-silico trials also with the existing standards applied in real clinical trials such as CDISC, CDASH?

S: Yes, if we plan to use this data in a regulatory environment the regulators are more familiar with these standards. But in practical terms not sure how easy this will be as some of the model parameters are not going to easily fit.

Question: Are there any standards used in the area of virtual cohorts?

W: We have physiological-based models or physics-based models, and for that there are a lot of guidelines e.g. the FDA has a lot of work in this field. They call it verification, validation and uncertainty quantifications and you need to demonstrate such aspects before you apply your models. So, we have guidelines showing how in-silico models should be developed. But this does not apply specifically to virtual cohorts in the sense of individual virtual patients that are used for predicting surgical outcomes or diagnosis or prognosis. These guidelines are produced by Tina Morrison.

L: On the European side, there isn't really a standard for that. We have V&V40. There are currently exercises in Europe such as EUSTANDS4PM who submitted a proposal to ISO and it is now on the subcommittees to vote on data collection and use of modelling and simulation. The scope is a bit limited and might not encompass all of the official physiological-based models. Another initiative is also GSP that is driven by the Avicenna Alliance and VPHi.

Wrap-up and conclusions (Christian Ohmann, ECRIN)

Next steps forward:

- Explore possibilities of integration also with Python, Jupyter notebook, Galaxy. If needed make the links with the EOSC-Life project WP2 on standardised workflows.
- Within SIMCor we should more clearly define the interaction between the three components: The Virtual Research Environment, the Virtual Cohort Generator and the Statistical Analysis Environment. Which data are transferred, what comes back as response and how are the data archived.
- To explore legal requirements: what is really needed based upon available documents and guidances and come into contact with regulators to discuss with them expectations. If these discussions start early on then we can adapt accordingly within the SIMCor project and the other in-silico projects.
- Explore the use of data standards and whether standards applied in real world clinical trials can be used for in-silico trials as well (e.g. CDISC, CDASH). When gaps are observed, e.g. for the model side, see whether other standards exist or are under development.

S3 Why R, RMarkdown, and Shiny?

Why R, R Markdown, and Shiny?

R is a free and open-source programming language specifically designed for statistical data analysis, which may require the application of complex statistical models, the implementation of new statistical procedures, and sophisticated data visualisation functionality. R runs on almost any standard computing platform and operating system.

It is worth knowing that R is a language implemented as a dialect of the S system. S is a language developed at Bell Laboratories in the seventies by John Chambers and collaborators. S aimed to be an interactive statistical analysis language that does not require a programming background. The S language was further developed in the 1980s using functional programming. In 1998, S was honoured with the Association for Computing Machinery's Software System Award (ACM), a prestigious recognition in computer science. Previously, ACM awards were given to iconic software such as the UNIX system, the World Wide Web, and PostScript.

Over the past decade, R has emerged as the leading statistical software for data analysis. One of the key factors contributing to the success of the R project is the standardisation in the development of R packages, which significantly expands the capabilities of the R system. R packages not only offer the implementation of new statistical models but also facilitate the integration of R with other data science languages (e.g. Julia, JAGS, Stan, Python, C++), as well as additional features such as dynamic reporting and data manipulation
.
Our project strategy primarily involved using two key R packages: RMarkdown (1), and Shiny (2). The RMarkdown package enables the development of documents that include R scripts, known as "R chunks", within an RMarkdown document. These documents can be rendered into reports in various formats, such as PDF, HTML, or Word.

This approach promotes replicability in statistical analysis because once a data set is generated, the entire workflow and results can be shared transparently with stakeholders, including researchers and regulatory bodies. Additionally, the RMarkdown documents can be used as templates for tasks such as creating statistical reports or developing statistical analysis plans.
On the other hand, the Shiny package provides the functionality to build interactive web applications with R. The package offers automatic "reactive" binding between inputs and outputs and extensive prebuilt widgets. Building a web application with Shiny allows end-users to explore, manipulate, and visualise data in real time. This functionality is crucial for stakeholders with varying technical

backgrounds, enabling them to interact with the simulation results without requiring R programming expertise.

In this way, the combination of R with R Markdown and Shiny provides a user-friendly ecosystem for planning, analysis, and reporting in silico clinical trials within a replicable research environment.

References

1. Xie, Y., Allaire J.J., Gurlemund, G. R Markdown: The Definitive Guide. Chapman and Hall/CRC, Boca Raton, Florida. https://bookdown.org/yihui/rmarkdown (2023)..

2. Chang, W. et al.: shiny: Web Application Framework for R. R package version 1.9.1.9000, https://github.com/rstudio/shiny, https://shiny.posit.co/ (2024).

S4 User stories

Title	ID	User story	Assumption	Comment
Import of data file(s)	SIMCor#1new	As a researcher I want to import data file(s) from external into the R- Statistical Environment. The procedure should at least support CSV format.	There should be a button with “import virtual dataset”, respectively “import real dataset”, allowing import of data file(s).	In future versions import of more file types should be possible.
Structure of imported data file(s)	SIMCor#2new	As a researcher I want to see a list of the variables of the imported data file(s), together with some basic statistics.	After import of a data file(s), a list of the variables contained in the data file should be presented automatically, including the number of datasets in the data file(s) and some basic statistics.	
Analysis of imported data file(s)	SIMCor#3new	As a researcher I would like to perform different types of analysis with the imported data file(s).	If data file(s) have been imported a list of different analysis options appears, covering analysis techniques for “validation of a virtual cohort” and “application of validated cohorts”. For the validation module, this includes univariate, bivariate and multivariate techniques and for the application module, it covers one-group design, two-group design and sample size calculation.	In version 0.1.0, there are two different applications, which need to be uploaded separately (“validation of virtual cohorts”, “application of validated cohorts”). It is planned to integrate both modules into one application in future versions.
Menu-driven application	SIMCor#4new	The application should be largely menu driven.	Individual analysis techniques implemented in the R-shiny application should be applicable via selection from a menu.	

Report	SIMCor#5new	As a researcher I should be able to get a standardized report of the data files used, the variables and algorithms specified and the results<br>of the analysis.	If an analysis has been performed, a button “report” can be clicked, and a standardized report is presented in pdf format. The report can be stored in the R-Statistical Environment.	A template for a standardized statistical report has been produced in R-markdown, but not yet integrated into the validation and application modules.<br>This feature will be implemented in the next version.
Assessing variability/ uncertainty of results	SIMCor#6new	As a researcher, I wish to assess the variability/ uncertainty of my results using bootstrapping techniques, to ensure the robustness and reliability of my findings.	Separately for the two modules (“validation of a virtual cohort”, “application of validated cohorts”) it should be possible to assess the variability/uncertainty of the results using simulations with random samples (bootstrapping). It should be possible to specify the input to this analysis in the application (e.g. size of the random sample, number of bootstrap samples, other parameters).	This feature is implemented in the validation module.<br><br>In the next version, this feature will be integrated into the application module. The aim is to assess variability of the statistical analysis by bootstrap methods.
Graphical presentation of results	SIMCor#7new	As a researcher I want to get graphical outputs of the analyses as much as possible.	For the results of the analysis graphical<br>techniques should be used. The figures should be automatically presented together with the numerical results and should cover boxplots, correlation heatmaps, survival curves, curves characterising the results of the bootstrap analyses, etc.	

Selectionof Clinical trial design	SIMCor#8new	As a researcher I want to define the clinical trial design applied to the data.	At the start of the application module, it should be possible to define the type of the trial. For the beginning two options should be available: 1-group design or 2-(parallel) group design.	The use of more study designs should be possible in future versions.
Specification of an outcome variable	SIMCor#9new	For the module “application of validated cohorts” the specification of an outcome variable should be possible.	In the application module, the specification of an outcome variable from the variables of an imported data file should be possible via selection from a list. In addition, it should be possible to specify the outcome variable type (qualitative, quantitative, time to event).	
Statistical analysis of the outcome variable	SIMCor#10new	As a researcher, I want to perform a statistical analysis of the outcome variable.	For different types of outcome variables (continues, qualitative, or time-to event), descriptive statistics are tabulated. In addition, statistical tests, and 95% confidence intervals are calculated for the parameter that summarises the statistical hypothesis of interest (e.g. mean in a 1-group design, difference of the means in a 2-group design, odds ratio for two-by-two tables).	
Planning of an in-silico trial	SIMCor#11new	The application module should support the planning of in-silico trial with sample size calculation.	For two-group comparisons sample size calculations should be possible. Initially, this should be implemented for a continuous outcome variable with a common standard deviation	Sample size calculation should be extended to qualitative and time-to- event outcome variables in future versions.

			between the groups. It should be possible to specify the necessary input (mean for group 1, mean for group 2, common standard deviation, significance level, power and hypothesis type) to perform the calculation. Some functionality for calculation of variability/uncertainty of sample size calculation should be made available (bootstrapping).	
User manual	SIMCor#12new	A structured and clear user manual with hypertext links to additional information should be provided.	At the start of the application, access to the user manual should be possible via a link.	
Help function (explanatory text)	SIMCor#13new	For all relevant functionalities access to context-sensitive explanatory information should be available.	If a functionality has been selected, there should be a link to explanatory information.	

S5 General model for R-statistical environment

SIMCor: General model for R-statistical environment

Authors: C. Ohmann, T. Khorchani (ECRIN),
P.E. Verde (Heinrich Heine University and University Hospital Düsseldorf)

Date: 7 December 2023

Version: Version 2, draft

Table of content

1.General aspects 2
2.Validation of virtual cohorts 2
2.1 Univariate (marginal) distributions of the variables between real and synthetic data 3
2.2 Bivariate correlations between variables of real and synthetic data 3
2.3Multivariate comparison of variables characterising the real and synthetic data 4
2.4 Analytical techniques taking uncertainties into consideration 4
2.5 Predictive model performance 4
3.Application of validated cohorts in in silico clinical trials 5
3.1One-group assessment 5
3.2Two-group comparison 6
4.References 7
5.**Appendices** 7
5.1 Multivariate q-q-plot to compare distributions between virtual cohorts and clinical datasets 7
5.2Bootstrap methods to compare distributions between virtual cohorts and clinical datasets ... 8
5.3 Study Protocol: In silico cohort 8
5.4 Statistical designs and sample size calculations for in silico trials 8

This document describes the analytical techniques to be implemented in the R-statistical environment. It does not cover the functionalities around management and import of virtual and real datasets and not the exports and reports generated from the statistical analysis. The work was performed by ECRIN and a subcontracted partner from the University of Düsseldorf within WP10 of SIMCor.

1. General aspects

Validation and application of virtual cohorts is related to the Context of Use (CuO) and the Question of Interest (QoI). Definitions for these terms are taken from the FDA Guidance on "Assessing the credibility of computational modelling and simulation in medical device submissions" (1).

COU: "a statement that defines the specific role and scope of the computational model used to address the question of interest"
QoI: "the specific question, decision, or concern that is being addressed"

Another term of relevance in this context is Quantity of interest (QoI) (1):
Quantity of interest: "the calculated or measured result from a computational model or comparator, respectively.

Example (taken from (1)):
COi: "Combine computational modeling predictions and empirical fatigue testing observations to estimate device fatigue safety factors under anticipated worst-case radial loading conditions,"
QoI: "Is the device resistant to fatigue fracture under anticipated worst case radial loading conditions?"

For models used in silico device testing or in silico clinical trials, the COU should describe how the model will be used in a simulation study to address the QoI. The QoI defines the specific and concrete question related to the CoU. As such CoU and CoI are prerequisites for any kind of validation or application activity directed at virtual cohorts or in silico trials.

CoU and QoI belong to the metadata of a virtual cohort and should be stored with other metadata in the VRE. So, the CoU and the QoL are already predefined when importing the virtual cohort dataset into R-statistical environment. CoU and QoI are essential elements for the scope and role of the computational model and the specific question addressed. This information is recorded as text and linked to the metadata of the virtual cohort. If no information is documented for CoU and QoI, this will be marked as "missing" in the R-statistical environment. The analytical techniques to validate and apply specific virtual cohorts are not directly linked to the CoU and the QoI but this information is included at the beginning of the statistical report to allow proper interpretation of the statistical results.

2. Validation of virtual cohorts

Validation is the "the process of determining the degree to which a model or a simulation is an accurate representation of the real world. This is different from verification, which is defined as "the process of determining that a computational model accurately represents

the underlying mathematical model and its solution from the perspective of the intended uses of modelling and simulation”. (1)

The R-statistical environment does not deal with verification and is only concerned with validation and applicability of virtual cohorts.

Validation is generally demonstrated by comparing the computational model predictions with the results from the comparator (2). Here, the comparator is defined as the test data that are used for validation, which may be data from bench testing or in vivo studies. In the R-statistical environment only in vivo studies (clinical studies or animal experiments) will be covered.

Model calibration evidence is not validation evidence because it is not testing of the final model against data independent of model development; instead, model parameters are calibrated (whether optimized or manually tuned) to minimize the discrepancy between model results and data (1). Validation in the R-statistical environment covers different analytical techniques of comparison between real and synthetic data. Prerequisite for any validation activity is similarity of the structure of the virtual cohort and the real data set.

The measured QoIs of the validation activities are not always identical to the QoIs for the CoU because the QoIs for the CoU are not always directly measurable, might not be measured without unduly perturbing the intended test conditions, and/or might not be obtained within acceptable ranges of uncertainty and error. Therefore, the measured QoIs of the validation activities may be surrogates for the QoIs for the CoU, with varying degree of applicability (2).

Therefore, the QoI for the validation activities may be different from the general QoI. The QoI for the validation activities must be selected from the variables characterising the imported dataset (one or more variables are possible) and has links to some of the statistical techniques to be applied in the R-statistical environment. It is documented together with the statistical analysis scripts and the results of the analysis and is included in the report.

2.1 Univariate (marginal) distributions of the variables between real and synthetic data

From the imported data sets all, several or one specific variable are selected and separately for the virtual and real datasets descriptive statistics are calculated and presented:

- Mean value, median value, standard deviation, interquartile range, range for quantitative variables
- Frequencies for qualitative variables
- Scatter plots of combinations of variables

The results are presented as tables with variables as rows and virtual and real data metrics as columns. In addition, the results are shown as box plots.

2.2 Bivariate correlations between variables of real and synthetic data

Here, separately for the real and synthetic dataset, bivariate correlations between the variables are calculated. The idea is to compare the correlations within the two cohorts.

- Spearman correlation coefficients between all features (separately for real and virtual data)

The results are graphically displayed as so-called heatmaps. Correlation heatmaps are a type of plot that visualize the strength of relationships between numerical variables. Correlation plots are used to understand which variables are related to each other and the strength of this relationship (6).

2.3 Multivariate comparison of variables characterising the real and synthetic data

To evaluate the compatibility of the virtual cohort with the real data, a multivariate comparison between the n-dimensional distributions of the features of both cohorts can be performed in the R-statistical environment. The following test will be used:

- Quantile-Quantile plot between the synthetic and the real data after multivariate standardization of each data sets. Multivariate standarization is performed by 1) subtracting the vector of means to each vector data point and 2) scaling by using the inverse of the variance covariance matrix. The resulting standardized quantity is a quadratic form. The methodology is explained in Appendix 1 (Multivariate q-q-plot to compare distributions between virtual cohorts and clinical datasets).

2.4 Analytical techniques taking uncertainties into consideration

In this validation approach, the stochastic results of the model (virtual dataset) and experiment (real dataset) are plotted together on a cumulative density function (CDF) plot for a variable of interest (3). The uncertainties in the model (due to input uncertainties and numerical uncertainties) and experiment (due to measurement system uncertainty and specimen-to-specimen variability) are represented in the two distributions. Any discrepancy in the two curves is therefore considered to be a manifestation of model form uncertainty. The uncertainty of the model is represented as an area around the cumulative density function and compared with the cumulative density function of the test data set. The area metric is defined by the area between the model (and its uncertainty) and experimental results from the real dataset.

In the R-statistical environment the generation of bootstrap samples to get an estimate of the variability and uncertainty is proposed. This is done via resampling of the virtual data and by comparing the distributions generated with the real data set. Here, 95% confidence bounds for the density function of the virtual data are calculated. A bootstrap $p - value$ can be calculated from the number of times that the density of the real data is out of the 95% confidence bounds of the bootstrap analysis. The results are graphically displayed as (cumulative) density functions. The methodology is described in Appendix 2 (Bootstrap methods to compare distributions between virtual cohorts and clinical datasets).

2.5 Predictive model performance

The development of predictive models plays a major role in the application of virtual cohorts. Example: A manufacturer develops a computational model-based tool that predicts if a patient will respond positively to proposed therapy and validates the predictive capability of the tool by performing a clinical trial and computing adequate statistical measures. To support the development of such models, a dependent variable from the list of all variables in the dataset must be specified and a predictive model based on independent variables from the dataset is constructed. Here different techniques can be used, such as logistic regression or multiple linear regression. In R it is possible to cover different techniques with one function (generalized linear models: glm() function R). The development of predictive models is not a task delt within the R-statistical environment but is performed by virtual cohort designers.

Of interest in the R-statistical environment is the validation of predictive models. This aspect deals with individual-level comparisons between model predictions and an independent clinical dataset (1). In the first version of the R-statistical environment this kind of analysis is not included. If additional resources will be available, the extension of the package to this kind of analysis will be taken into consideration.

3. Application of validated cohorts in in silico clinical trials

Applicability is defined as " the relevance of a credibility assessment activity (e.g., validation activities) to support the use of the computational model for a context of use" (1). The applicability of the validation activities is governed by two factors: the relevance of the QoI used in the validation to the QoIs of the CoU, and the relevance of the validation conditions relative to those of the CoU (2).

The applicability is given, provided the relevance of the QoI as well as the relevance of the validation activities on the CoU has been shown (4) or in other words, whether the validation evidence provided are relevant within the CoU of the model (5). Applicability is a prerequisite for application of validated cohorts in silico clinical trials.

But even when there are enough data to achieve sufficient statistical power, a more general problem of applicability remains. To be useful, a model should be able to make predictions for input values that are different from those used to assess its accuracy; but we do not know the predictive accuracy of the model for those new inputs. Considerations on the general regularity of physical quantities, and about the assumption that model accuracy should degrade smoothly in the sense that predictions made for similar inputs should present similar predictive accuracy, allow to assume that a degree of extrapolation is possible, in the sense that the model can be considered reliable even when used to predict for inputs different from those observed in the clinical validation cohort.

The following analytical techniques are applied in the R-statistical environment:

3.1 One-group assessment

Here only one validated virtual cohort is analysed with respect to univariate descriptive statistics. This covers different types of variables, such as prognostic factors, interventions, and outcomes. From the imported virtual cohort all, several or one specific variable are selected, and the following descriptive statistics are calculated and presented:

- Mean value, median value, standard deviation, interquartile range, range for quantitative variables
- Confidence intervals for individual variables

- Frequencies for qualitative variables

The results are presented as tables with variables as rows and descriptive statistics in columns. In addition, the results are shown as box plots.

In addition, an assessment of the variability will be performed by enabling random sub-sampling from the virtual cohort. The results from this assessment are presented separately for each subsample and are summarised in a graphical display.

Bivariate dependencies between certain variables in the virtual cohort can be analysed with the following methods:

- Spearman correlation coefficients between selected features
- Relative risk (for certain outcomes)

Optionally, if resources are available, this will be completed by the possibility to perform multivariate analysis for prediction of outcome variables.

Here the "generalized linear model" glm() function R will be used. This function covers, binary outcomes (logistic regression), continues outcomes (multiple linear regression), counting outcomes (Poisson regression) and so on. So, several scenarios are covered with one function. In addition, step-wise variable selection can be implemented (e.g. function stepAIC() in R).

3.2 Two-group comparison

The major use case for a two-group comparison of validated virtual cohorts is an in-silico trial comparing two medical devices. For the two-group design two validated virtual cohorts with the same structure and variables are needed, only differing by the type of intervention. For the analysis it should be possible to specify interesting outcome variables. The analysis should include the usual descriptive statistics but also different statistical tests for comparing the two groups.

With respect to descriptive statistics, the following metrics are foreseen:

- Mean value, median value, standard deviation, interquartile range, range for quantitative variables
- Confidence intervals for individual variables
- Frequencies for qualitative variables

The results are presented as tables with variables as rows and descriptive statistics in columns. Descriptive statistics is performed, and the results displayed separately per group. In addition, the univariate results are shown as box plots, again separately for both groups.

Furthermore, different statistical tests for comparing the two groups are included:

- T-test, Wilcoxon-test for quantitative variables
- Chi-square, Fisher-test for qualitative variables

The application should also provide possibilities to perform:

- Power considerations
- Analysis of effect sizes

Dependent on the available resources, the following options for selection of clinical trial alternatives are considered:

- Analysis method (superiority, non-inferiority, equivalence)
- Hypothesis (2-tailed, 1-tailed)
- Type 1 error

There are more aspects of clinical trials, which could also be of interest for in-silico clinical trials. These aspects must be discussed more in detail before an implementation in the R-statistical environment can be recommended and considered. These aspects cover:

- Trial design (fixed, adaptive, sequential)
- Drop-out rate
- Interim analysis/stop criteria
- Analysis (intention to treat, per protocol)

In Appendix 5.3 "Study Protocol: In silico cohort" is included and in appendix 5.4 "Statistical designs and sample size calculations for in silico trials" is described.

4. References

1. FDA: Assessing the credibility of computational modeling and simulation in medical device dubmissions. Draft guidance for industry (December 2021)
2. ASME V&V 40-2018: Assessing credibility of computational modeling through verification and validation: Application to medical devices (2018)
3. Bodner, J., Kaul, V.: A framework for in silico clinical trials for medical devices using concepts from model verification, validation, and uncertainty quantification (VVUQ). Proceedings of the ASME 2021, May 19-20, 2021)
4. Aldieri A., Curelli, C., Szyszko, j. A., La Mattina, A.A., Viceconti, M.: Credibility assessment of computational models according to ASME V&V40: Application to the Bologna Biomechanical Computed Tomography solution. Computer methods and Program 240: 107727 (2023)
5. Visconti, M., Juarez, M.A., Curelli, C. Pennisi, M.,Russo, G., Pappalardo, F.: Position paper: Credibiility of in silico trial technologies: A theoretical framing. IEEE Journal of Biomedical and Health Informatics 24, 2020
6. Verstraeten, S., Hoeijmakersb, M., Pim Tonino, P., c, Jan Brüning, J., Capelli, C., van de

   Vosse, F., Huberts, W.: Generation of synthetic aortic valve stenosis geometries for in silico trials (submitted, 2023)

5. Appendices

5.1 Multivariate q-q-plot to compare distributions between virtual cohorts and clinical datasets

5.2 Bootstrap methods to compare distributions between virtual cohorts and clinical datasets

5.3 Study Protocol: In silico cohort

5.4 Statistical designs and sample size calculations for in silico trials

5.1

Multivariate q-q-plot to compare distributions between virual cohorts and clinical datasets

Pablo Emilio Verde

23 Oktober 2023

Contents

Multivariate q-q-plot: **2**

Real and synthetic data example **3**

Multivariate q-q-plot:

The aim is to compare the empirical probability distributions of two data sets: a real clinical data y_r and a virtual cohort data y_v produced by an algorithm.

Me assume that the dimension of y_r is $p \times n_r$ and the dimension of the virtual cohort data y_v is $p \times n_v$.

The mean and the variance covariance matrix of y_r are:

$$E(y_r) = \mu_r \quad \text{with dimension} \quad (p \times 1),$$

and

$$Var(y_r) = \Sigma_r \quad \text{with dimension} \quad (p \times p).$$

Similarly, the the mean and the variance covariance matrix of y_v are:

$$E(y_v) = \mu_v \quad \text{with dimension} \quad (p \times 1),$$

and

$$Var(y_v) = \Sigma_v \quad \text{with dimension} \quad (p \times p).$$

In order to reduce the dimensionality of the multivariate comparison we compare the standardized observation calculated with the following quadratic forms:

$$q_r = y_r^T \times \Sigma^{-1} \times y_r,$$

and

$$q_v = y_v^T \times \Sigma^{-1} \times y_v.$$

The quantities q_r and q_v have dimension 1. Moreover, if the multivariate normality of y_r and y_v hold then we have that

$$q_r \sim \chi^2_p \quad \text{and} \quad q_v \sim \chi^2.$$

To compare the multivariate distributions of y_r and y_v, we compare the empirical quantiles of the univariate distributions of q_r and q_v. This comparison is performed with a qq-plot.

Real and synthetic data example

```r
library(readxl)
shapeFeatures_real <- read_excel("shapeFeatures_real.xlsx")
```

```r
library(readxl)
shapeFeatures_virtual <- read_excel("shapeFeatures_synthetic.xlsx")
```

We calculate the multivariate standardized outcomes for the virtual data:

```r
# The sample size is
n.virtual = dim(shapeFeatures_virtual)[1]

# Calculate the mean and the variance covariance matrix
 mean.virtual = colMeans(shapeFeatures_virtual)
Sigma.virtual = var(shapeFeatures_virtual)

# Calculate the inverse of the variance covariance matrix

Sigma.virtual.inverse = solve(Sigma.virtual)

# Calculate the quadratic form:
# The function within the apply center the columns and calculate the quadratic form.

q.virtual = apply(shapeFeatures_virtual, 1,
                      function(x, mean.virtual, Sigma.virtual.inverse)
                      {
                      t(x-mean.virtual) %*% Sigma.virtual.inverse %*% (x - mean.virtual)},
                      mean.virtual, Sigma.virtual.inverse)
```

The same calculations for the real data:

```r
# The sample size is
n.real = dim(shapeFeatures_real)[1]

# Calculate the mean and the variance covariance matrix
 mean.real = colMeans(shapeFeatures_real)
Sigma.real = var(shapeFeatures_real)

# Calculate the inverse of the variance covariance matrix

Sigma.real.inverse = solve(Sigma.real)

# Calculate the quadratic form:
# The function within the apply center the columns and calculate the quadratic form.
```

```
q.real = apply(shapeFeatures_real, 1,
               function(x, mean.real, Sigma.real.inverse)
               {
               t(x-mean.real) %*% Sigma.real.inverse %*% (x - mean.real)},
               mean.real, Sigma.real.inverse)
```

Comparison of quadratic form vs. a χ^2_p distribution:

```
# degrees of freedom are the number of columns of the original data
p = dim(shapeFeatures_real)[2]
max.q.virtual = max(q.virtual)
max.q.real = max(q.real)

par(mfrow = c(1,2))
hist(q.virtual, breaks = 40, probability = TRUE, ylim = c(0, 0.14))
curve(dchisq(x, df = p-1), from = 0, to = max.q.virtual, lwd = 2, col = "blue", add = TRUE)

hist(q.real, breaks = 40, probability = TRUE, ylim = c(0, 0.14))
curve(dchisq(x, df = p-1), from = 0, to = max.q.real, lwd = 2, col = "red", add = TRUE)
```

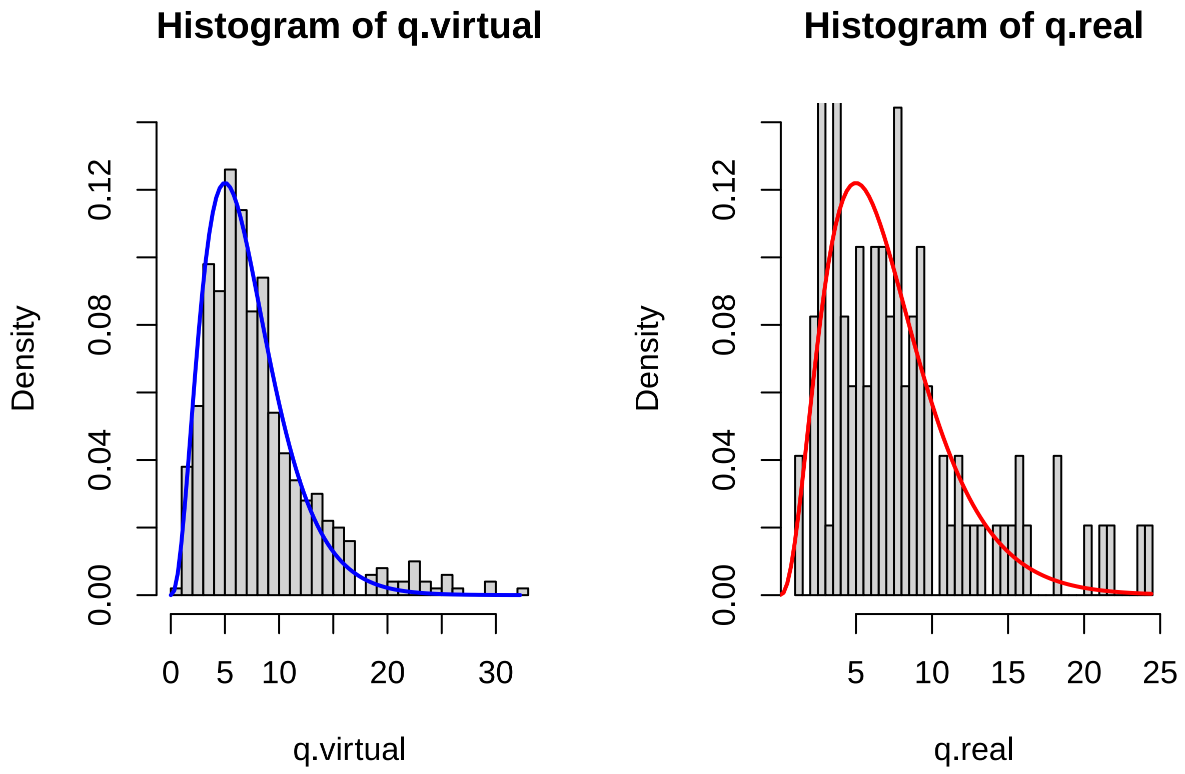

```
par(mfrow = c(1,1))
```

Finally, the qq-plot to compare the virtual and the real data:

```
qqplot(q.virtual, q.real)
abline(a = 0, b =1, lwd = 2)
```

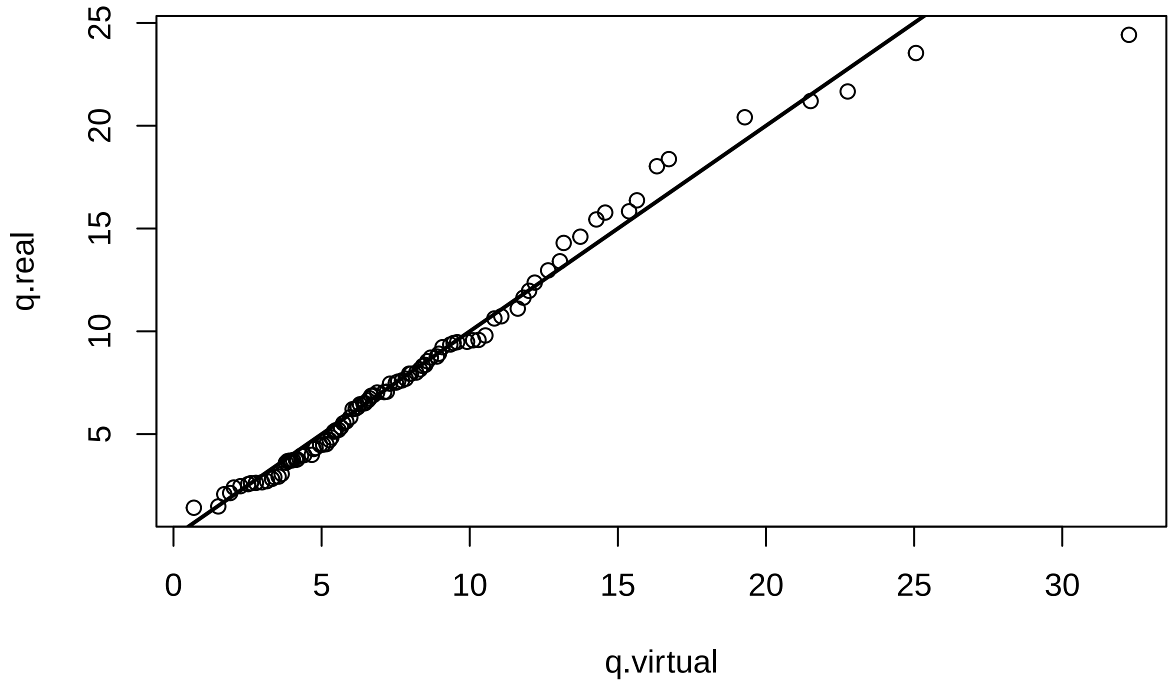

5.2

Bootstrap methods to compare distributions between virual cohorts and clinical datasets

Pablo Emilio Verde

15 September 2023

Contents

Proposed Bootstrap method for univariate measurements **2**

Real and synthetic data examples **3**

Real shapes data 3

Bootstrap comparison of the density function 3

Features comparison virtual vs real **8**

Multivariate comparisons **9**

Proposed Bootstrap method for univariate measurements

The aim is to compare the probability distributions of two data sets: a real clinical data y^r and a virtual cohort data y^v produced by an algorithm.

We propose to generate bootstrap samples, by resampling the virtual data y^v and compare the distributions generated with the fixed y^r. The bootstrap comparison has to be performed by fixing the data sets: $n_r = n_v$.

We assume that each observation is univariate and independently distributed and we denote these two data sets as:

$$y_1^r \ldots, y_{n_r}^r \sim P^r$$

and

$$y_1^v \ldots, y_{n_v}^v \sim P^v.$$

In addition, we assume that we do not have directly access to the algorithm that produced y_r, but only a realization of the virtual cohort. Moreover, the real data y^r is a **validation data** that has not been used to build the model P^v that simulates y_r.

The central difficult is that we are comparing a **directly measured** data y^r from patients, with an **indirect generated** data y^v that aims to mimic y^r. The main difference between the two data sets is the **amount of information** that they contain.

This feature can be describe by the relationship between the sample sizes n_r and n_v. For a sample size of real measurement n_r the effective sample size of a virtual cohort is $n_v = \alpha \times n_r$, where α is an **uncertainty discount factor** between zero and one. Thus, if $\alpha = 0.10$ a virtual observation worth 0.1 of a real one.

If we handle the virtual data y^v as a prediction of the true data y^r, we expect that the prediction will have more variability and we can choose α as

$$\alpha = \frac{Var(y_r)}{Var(y_v)}.$$

We use this technique in the example below.

We use this relationship between the sample sizes to generate the bootstrap samples from the virtual data y^v. Thus, the bootstrap samples are sub-samples with replacement of size $m = [\alpha \times n_r]$. For example, $n_r = 100$ and we choose $\alpha = 0.70$, the sample size of the bootstrap sample is $m = 70$.

Real and synthetic data examples

Real shapes data

```
library(readxl)
shapeFeatures_real <- read_excel("shapeFeatures_real.xlsx")
```

```
library(readxl)
shapeFeatures_virtual <- read_excel("shapeFeatures_synthetic.xlsx")
```

We choose the variable D_lvot as an example. We must take the same length between real and virtual data sets! We take

$$n_r = n_v = 90.$$

```
y.virtual = shapeFeatures_virtual$D_lvot[1:90]
y.real    = shapeFeatures_real$D_lvot[1:90]
```

Bootstrap comparison of the density function

```
plot(density(y.virtual), lwd = 2, col = "blue", ylim = c(0, 0.2),
     xlab = "Variable: D_lvot",
     main = "Comparison: Real vs. Virtual Cohort")
lines(density(y.real), lwd = 2, col = "red")
legend(30,0.15, legend=c("Real", "Virtual"), col=c("red", "blue"), lwd=c(3,3))
```

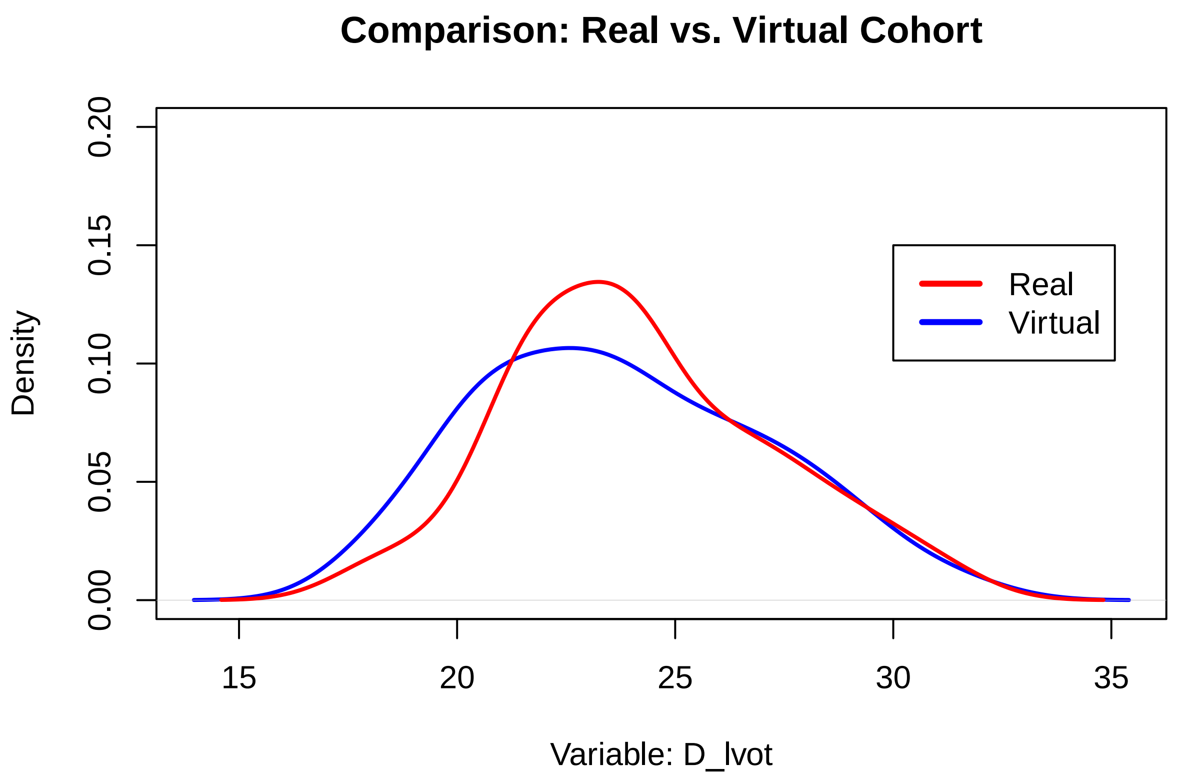

The sample size is:

```
n = length(y.virtual)
n
```

```
## [1] 90
```

We choose α as the ratio of the variances:

```
alpha = var(y.real)/var(y.virtual)
alpha
```

```
## [1] 0.8321351
```

and the size of the bootstrap samples as:

```
m = round(n*alpha,0)
m
```

```
## [1] 75
```

We choose the number of bootstrap samples B as:

```
B = 2000
```

We perform the bootstrap samples

```
set.seed(1509)
boot.mat.x = rep(0, 512*B)
dim(boot.mat.x) = c(B, 512)

boot.mat.y = rep(0, 512*B)
dim(boot.mat.y) = c(B, 512)



for(b in 1:B)
{
  y.star = sample(y.virtual, size = m, replace = TRUE)
  boot.mat.x[b, ] = density(y.star)$x
  boot.mat.y[b, ] = density(y.star)$y
}
```

Here we plot the real and the virtual data with 50 bootstrap samples from the virtual data:

```
plot(density(y.virtual), lwd = 2, col = "blue", ylim = c(0, 0.2),
     xlab = "Variable: D_lvot",
     main = "Comparison: Real vs. Virtual Cohort")
lines(density(y.real), lwd = 2, col = "red")
legend(30,0.15, legend=c("Real", "Virtual", "Bootstrap"),
       col=c("red", "blue", "grey"), lwd=c(3,3))



for(b in 1:5)
{
  y.star = sample(y.virtual, size = n, replace = TRUE)
  lines(density(y.star), col="grey", lty = 3)
}
```

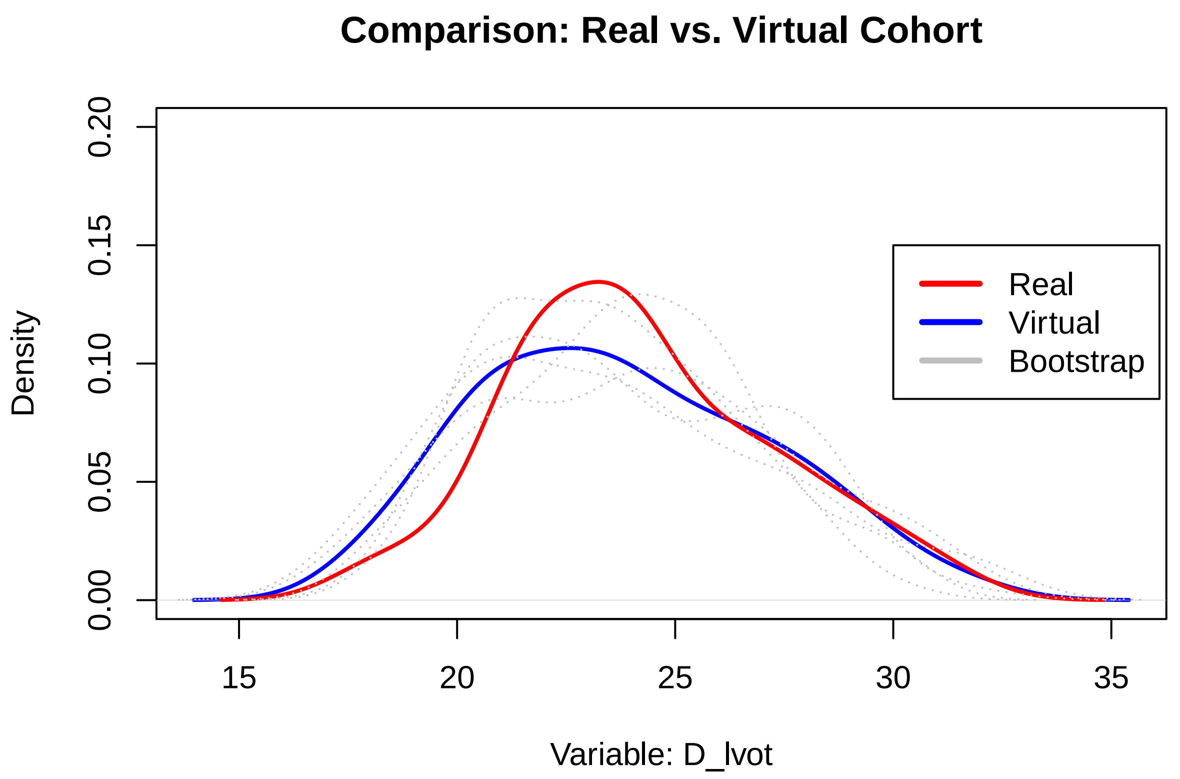

Here we calculate the 95% confidence bounds for the density function of the virtual data:

```
plot(density(y.virtual), lwd = 2, col = "blue", ylim = c(0, 0.2),
     xlab = "Variable: D_lvot",
     main = "Comparison: Real vs. Virtual Cohort")
lines(density(y.real), lwd = 2, col = "red")

x.boot = apply(boot.mat.x, 2, mean)

y.boot.95 = apply(boot.mat.y, 2, quantile, 0.975)
points(x.boot, y.boot.95, type = "l", lty = 2, lwd = 2)

y.boot.05 = apply(boot.mat.y, 2, quantile, 0.025)
points(x.boot, y.boot.05, type = "l",lty = 2, lwd = 2)

legend(28,0.18, legend=c("Real", "Virtual", "Bootstrap 95%"),
       col=c("red", "blue", "black"), lwd=c(3,3,2), lty = c(1,1,2))
```

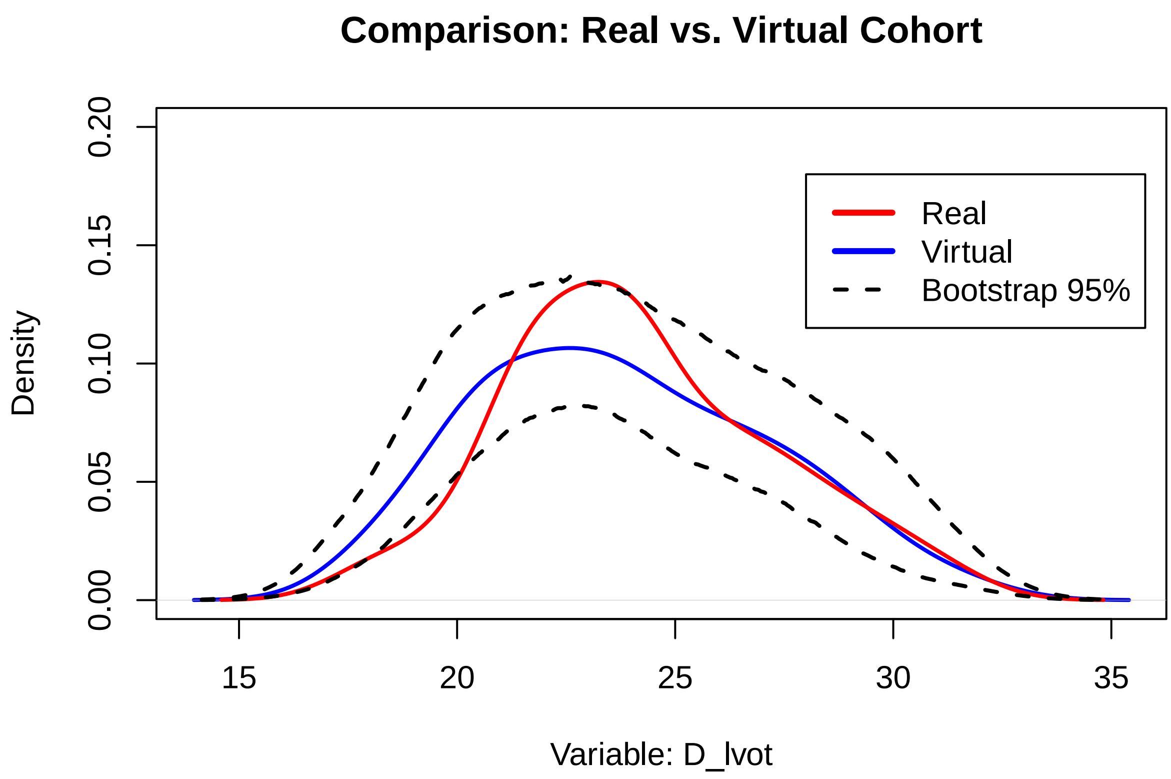

A bootstrap $p-value$ can be calculated from the number of times that the density of the real data is out off the 95% confidence bounds.

This statistical test can be calculated as follows:

```
n.upper = sum(density(y.real)$y> y.boot.95)

n.lower = sum(density(y.real)$y< y.boot.05)

n.upper
```

```
## [1] 14
```

```
n.lower
```

```
## [1] 25
```

```
x.length = length(x.boot)
```

The bootstrap $p-value$ is:

```
# P-value
0.5*n.upper/x.length + 0.5*n.lower/ x.length
```

```
## [1] 0.03808594
```

This p-value is less than 0.05, so the virtual cohort does not mimic the true data set.

Features comparison virtual vs real

Under construction

We can use the bootstrap samples to compare specific features of the data. For example, we can compare if the minimum or the maximum values of the virtual cohort mimics the corresponding values in the real data.

```
# Features: Min, Max

min.real = min(y.real)
max.real = max(y.real)

min.star = rep(0, B)
max.star = rep(0, B)

for(b in 1:B)
{
  y.star = sample(y.virtual, size = round(n*alpha,0), replace = TRUE)
  min.star[b] = min(y.star)
  max.star[b] = max(y.star)
}


# p values

sum(min.star<min.real)/B
```

```
## [1] 0.569
```

```
sum(max.star>max.real)/B
```

```
## [1] 0.5545
```

```
#Comparison plots

par(mfrow = c(1,2))

# min
hist(min.star, col = "blue", breaks = 50 )
abline(v = min.real, lty = 2, lwd = 4)

# max
hist(max.star, col = "red", breaks = 50 )
abline(v = max.real, lty = 2, lwd = 4)
```

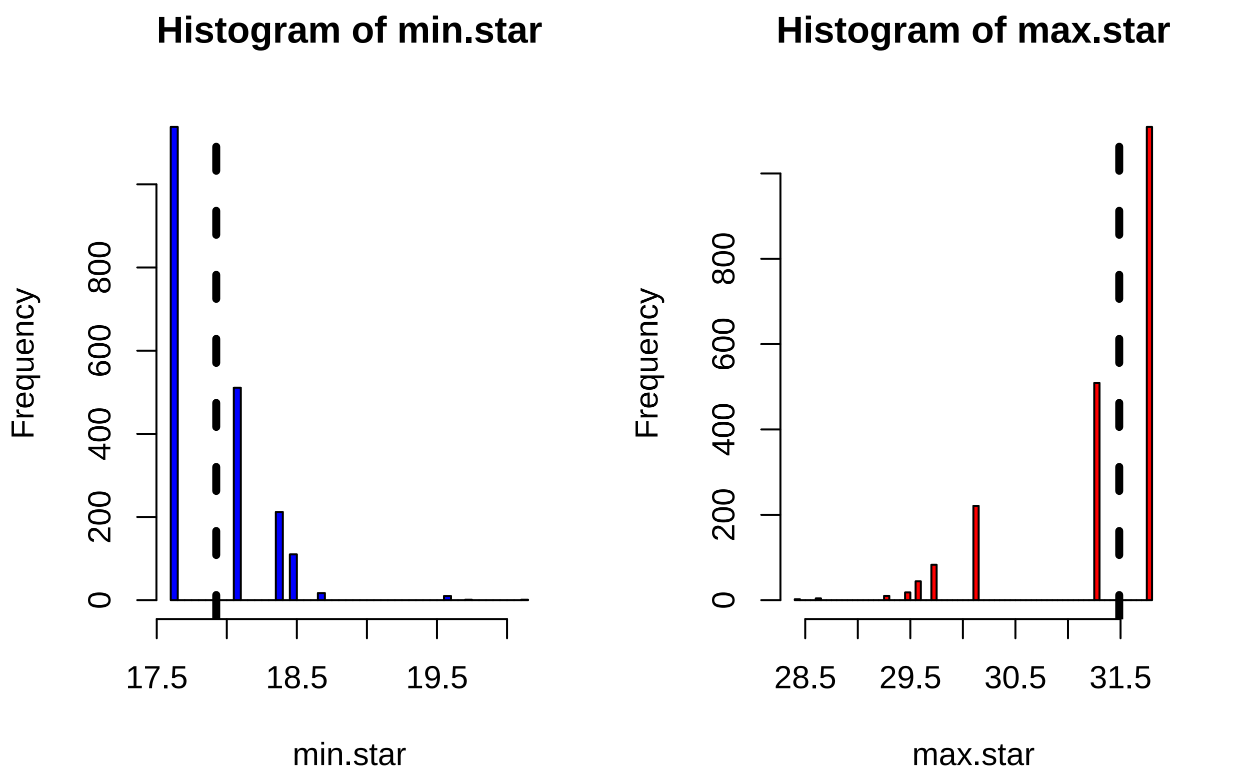


```
par(mfrow = c(1,1))
```

Multivariate comparisons

Under construction

5.3

Study Protocol: In Silico Cohort (Draft V01)

Author Name

21 April 2023

Contents

1 Introduction and background of the study **2**

2 Statements of hypothesis and aims of the study **2**

2.1 Demographic characteristics of the target cohorts 2

2.2 Clinical primary endpoints 2

2.3 In silico primary endpoints 2

2.4 Clinical secondary endpoints 2

2.5 In silico secondary endpoints 2

3 Transfering functions to link clinical and in silico endpoints **2**

3.1 Transfering functions for primary outcomes 2

3.2 Transfering functions for secondary outcomes 2

4 Design of the computer simulation generated data **3**

4.1 Methods used for generating 3

4.2 Sample size determinations 3

4.3 Methods for validation 3

5 Statistical methods for analysis **3**

5.1 Summary and descriptive statistics 3

5.2 Statistical analysis of primary endpoints 3

5.3 Statistical analysis of secondary endpoints 3

1 Introduction and background of the study

2 Statements of hypothesis and aims of the study

2.1 Demographic characteristics of the target cohorts

2.2 Clinical primary endpoints

2.3 In silico primary endpoints

2.4 Clinical secondary endpoints

2.5 In silico secondary endpoints

3 Transfering functions to link clinical and in silico endpoints

3.1 Transfering functions for primary outcomes

3.2 Transfering functions for secondary outcomes

4 Design of the computer simulation generated data

4.1 Methods used for generating

4.2 Sample size determinations

4.3 Methods for validation

5 Statistical methods for analysis

5.1 Summary and descriptive statistics

5.2 Statistical analysis of primary endpoints

5.3 Statistical analysis of secondary endpoints

5.4

Statistical designs and sample size calculations for insilico trials

Version 1

Pablo Emilio Verde
Takoua Khorchani
Christian Ohmann

07 December 2023

Contents

1 Introduction **2**

2 General statistical concepts **2**

3 Example of sample size calculations for comparing two devices **4**

3.1 Continuous outcome variable and one sided hypothesis 4

3.2 Continues outcome variable and two sided hypothesis 4

4 Including uncertainty in the sample size calculations **5**

1 Introduction

In this document we cover the sample size calculations for:

- An insilico trial that assesses continuous outcome variables of two independent devices, say device A vs. device B.
- We present the statistical background for one-sided, and two-sided statistical hypothesis that are tested with t-tests procedures.

2 General statistical concepts

For each device, say device A and B, we denote by $y_{i,j}$ the simulated clinical outcome for a virtual patient i that belongs to the group j, i.e.:

$$y_{i,j} \equiv \text{Measurement of patient } i \text{ in group } j.$$

We have the following working assumptions:

- The number of simulated patients is the same in each group, i.e., for $j = A, B$ we have $n_A = n_B = n$.
- A transfer function has been used to map the engineering outcomes to a clinical outcome, and the transfer function does not add uncertainty in the simulation process. We will relax this assumption in the following sections by adding uncertainty in the assessment of the effect size.
- We can summarize the distributions of two clinical outcomes by their means, μ_1 and μ_2, and a common standard deviation σ.
- For example, if the clinical outcomes follows a normal distribution, then they can be simulated from the following distributions:

$$y_{i,j} \sim N(\mu_j, \sigma^2), \quad \text{for} \quad i = 1, \ldots, n \quad \text{and} \quad j = A, B.$$

- The aim of an insilico trial is to test the difference between the means, μ_1 and μ_2. This hypothesis can be formulated as:

$$H_0 : \mu_1 = \mu_2 \quad \text{vs.} \quad H_1 : \mu_1 \neq \mu_2. \tag{1}$$

The above hypothesis formulation (1) is called **two sided**, which means that we are only interested in the difference between μ_1 and μ_2.

If the aim of an insilico trial is to determine if $\mu_1 < \mu_2$, we say that the hypothesis is one sided, and we state:

$$H_0 : \mu_1 \geq \mu_2 \quad \text{vs.} \quad H_1 : \mu_1 < \mu_2. \tag{2}$$

In order to test a statistical hypothesis, we need to calculate the number of virtual patients n in each group. This sample size calculation involves the following components:

1. The *Effect Size* (ES) that measure **the degree to which the null hypothesis is false**. For a null hypothesis comparing the means of two independent groups, the EF is:

$$ES = \frac{|\mu_1 - \mu_2|}{\sigma}.$$

2. The probability of **Type I error**, α, which is the probability of rejecting the null hypothesis, when it is true. This is thinking we have found an effect where none exist. This is considered the more serious error. Our tolerance for **Type I error** is usually $\alpha = 0.05$ or lower.

3. The probability of **Type II error**, β, which is the probability of accepting the null hypothesis, when it is false. This is thinking there is no effect when in fact there is. Our tolerance for Type II error is usually $\beta = 0.20$ or lower. **Type II error** is **1 - Power**. If we desire a power of 0.90, then we implicitly specify a **Type II error** tolerance of 0.10.

3 Example of sample size calculations for comparing two devices

3.1 Continuous outcome variable and one sided hypothesis

- We assume that the continues outcome is our **primary outcome** and could represent, e.g., a quality of life measurement.
- Given that this a primary outcome, we calculate the sample size of the virtual cohort based on this variable.
- We take $alpha = 0.05$ **one sided test**, power $= 90\%$:
- We state that $\mu_1 = 10$, $\mu_2 = 20$, and $\sigma = 20$. This means that we assume an effect size $ES = 0.5$.
- We are interested in testing $\mu_2 > \mu_1$.

In, R notation this problem is describe as follows:

```
mean1 = 10
mean2 = 20

sd.common = 20

sample.size = power.t.test(delta = mean2-mean1,
                           sd = sd.common,
              type = "two.sample",
              sig.level = 0.05,
              power = 0.9,
              alternative = "one.sided")
sample.size
```

```
##
##      Two-sample t test power calculation
##
##               n = 69.19782
##           delta = 10
##              sd = 20
##       sig.level = 0.05
##           power = 0.9
##     alternative = one.sided
##
## NOTE: n is number in *each* group
```

In this insilico trial we need at least 69 virtual patients per group.

3.2 Continues outcome variable and two sided hypothesis

We can repeat the example assuming that the hypothesis is two sided, i.e., we are interested in testing if $\mu_1 \neq \mu_2$.

In, R notation this problem is describe as follows:

```
mean1 = 10
mean2 = 20

sd.common = 20

sample.size = power.t.test(delta = mean2-mean1,
                          sd = sd.common,
              type = "two.sample",
              sig.level = 0.05,
              power = 0.9,
              alternative = "two.sided")
sample.size
```

```
##
##      Two-sample t test power calculation
##
##               n = 85.03129
##           delta = 10
##              sd = 20
##       sig.level = 0.05
##           power = 0.9
##     alternative = two.sided
##
## NOTE: n is number in *each* group
```

In this insilico trial we need at least 85 virtual patients per group.

4 Including uncertainty in the sample size calculations

Sample size calculations are very sensitive to the setup of the effect size. In practice, this parameter is unknown, and in particular for insilico trials, the effect size could depend on the transfer function that is used to link the ingeniering outcomes to the clinical outcome.

On simple way to tackle this problem is to give a probability distribution to the effect size, and run the sample size calculations for a large number of scenarios.

We can apply this procedure for the **two-sided** hypothesis testing problem. We describe the uncertainty of the effect size by applying uniform distribution between 0.2 to 1:

$$ES \sim \text{Uniform}(0.2, 1)$$

In R notation, we run $B = 10,000$ scenarios as following:

```
B = 10000 # number of scenarios
lower.ES = 0.2
```

```r
upper.ES = 1

ES = runif(B, lower.ES, upper.ES) # Uniform distribution of the effect size

n.star = rep(B, 0)

for(b in 1:B)
{
  n.star[b] = sample.size = power.t.test(delta = ES[b],
                          sd = 1,
              type = "two.sample",
              sig.level = 0.05,
              power = 0.9,
              alternative = "two.sided")$n
}
```

The following figure shows the resulting distribution of the sample size n per group:

```r
hist(n.star, breaks = 50, main = "Sample size distribution", xlab = "n")
```

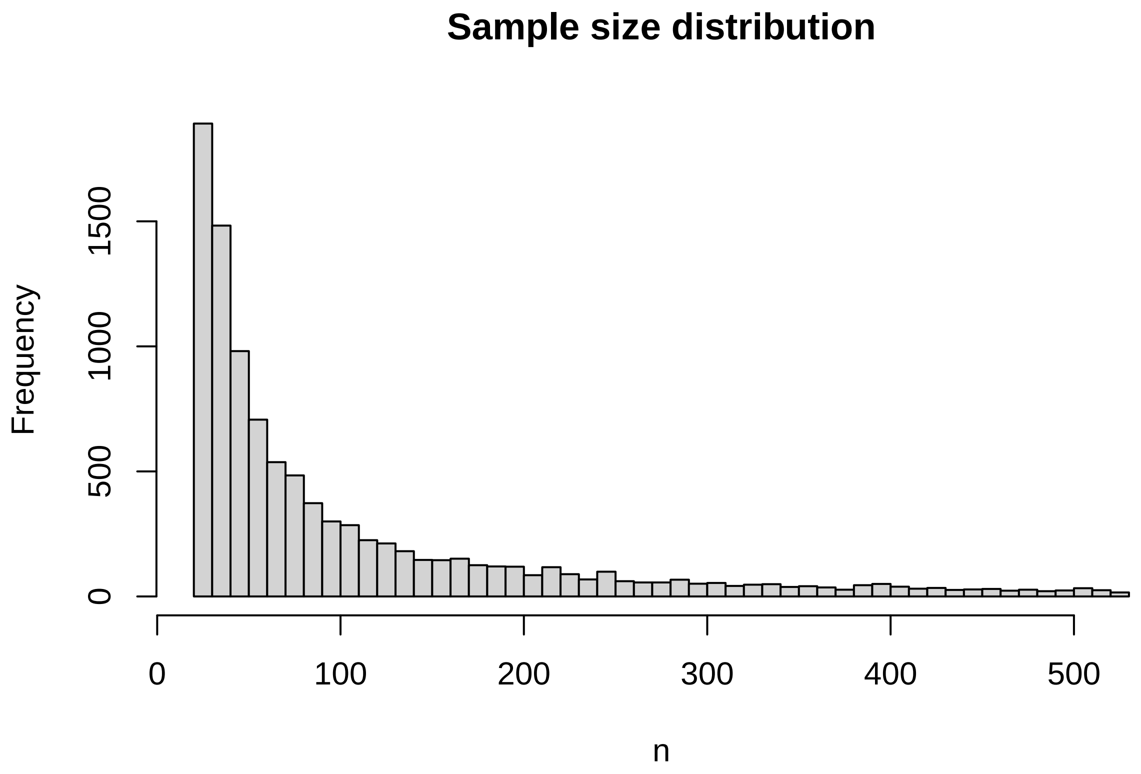

The distribution of the sample size has the following descriptive statistics:

```
summary(n.star)
```

```
##    Min. 1st Qu.  Median    Mean 3rd Qu.    Max.
##   22.03   33.45   58.94  105.21  130.88  526.04
```

The mean of the sample size distribution is $n = 106$, which shows an increase in the sample size when we add uncertainty in the calculations. Alternatively, we can use the third quantile (3rd Qu.) ,i.e., $n = 129$ as proposed sample size.

We can assess the power of the insilico trial by running again $B = 10000$ scenarios and fixing $n = 85$ (deterministic calculations), $n = 106$ (uncertain calculations, mean), and $n = 129$ (uncertain calculations, 3rd Qu.).

```
B = 10000 # number of scenarios
lower.ES = 0.2
upper.ES = 1

ES = runif(B, lower.ES, upper.ES) # Uniform distribution of the effect size

power.star.85  = rep(B, 0)
power.star.106 = rep(B, 0)
power.star.129 = rep(B, 0)

for(b in 1:B)
{
  power.star.85[b] = power.t.test(delta = ES[b],
                           sd = 1,
            type = "two.sample",
            sig.level = 0.05,
            n = 85,
            alternative = "two.sided")$power

  power.star.106[b] = power.t.test(delta = ES[b],
                           sd = 1,
            type = "two.sample",
            sig.level = 0.05,
            n = 106,
            alternative = "two.sided")$power

  power.star.129[b] = power.t.test(delta = ES[b],
                           sd = 1,
            type = "two.sample",
            sig.level = 0.05,
            n = 129,
            alternative = "two.sided")$power
```

```
}
```

The probability that the power < 0.9 under the three different sample sizes is:

```
sum(power.star.85 < 0.9)/10000 * 100
```

```
## [1] 37.09
```

```
sum(power.star.106 < 0.9)/10000 * 100
```

```
## [1] 30.72
```

```
sum(power.star.129 < 0.9)/10000 * 100
```

```
## [1] 25.35
```

If we do not include uncertainty in the sample size calculations, then we have a probability of 37% that the trial will be under-powered. If we increase the sample size of each group to $n = 129$ this probability is reduced to 25%.

S6: Development history of the application and R-packages integrated in the R-statistical environment

Development history of the application

1. Conceptualization

- **Activity:** Initial project ideation and alignment with objectives of the SIMCor project. Defined the scope for developing an R-statistical environment to support in-silico trials.
- **Key Outputs:**
  - Established goals: validation of virtual cohorts and application in in-silico trials.
  - Preliminary identification of tools and methods.
- **Date:** 2021

---

2. Preparatory Work

- **Activity:** Conducted workshops and surveys to evaluate tools and frameworks suitable for the project.
- **Key Outputs:**
  - Workshop (2021) to discuss the application scope.
  - Survey of existing statistical tools and R-packages for computational modeling.
  - Decision to develop a new R-(package) due to limitations in existing solutions.
- **Date:** 2021

---

3. Development of User Stories

- **Activity:** Creation and refinement of user stories to guide software development.
- **Key Outputs:**
  - Defined 13 user stories outlining features for the R-statistical environment.
  - Iterative updates based on discussions and evolving project needs.
- **Date:** 04/2023

---

4. Investigation of Statistical Tools and Methods

- **Activity:** Comprehensive evaluation of statistical tools and methodologies.
- **Key Outputs:**
  - Developed strategies for implementing statistical validation and application techniques.
- **Date:** 2023-2024

---

5. Development of the General Statistical Model

- **Activity:** Designed a comprehensive statistical model to validate virtual cohorts and conduct in-silico trials.
- **Key Outputs:**
  - Developed methods for one-, two-, and multivariate comparisons.
  - Designed approaches for variability and uncertainty assessments.
- **Date:** 10/2023

---

6. Conception of the Global Module

- **Activity:** Initial concept of a global module encompassing all functionalities.
- **Key Outputs:**
  - Conception for comprehensive module for validation and application of virtual cohorts.
  - Conception for initial integration of statistical methods.
- **Date:** 2023

---

7. Splitting of the Global Module

- **Activity:** Divided the global module into specialized modules for enhanced functionality.
- **Key Outputs:**
  - **Validation Module:** Focused on validating virtual cohorts using comparative analyses.
  - **Application Module:** Enabled application of validated cohorts in in-silico trials.
- **Date:** 10/2023

---

8. Programming Individual Modules

- **Activity:** Development and testing of individual modules for the application.
- **Key Outputs:**
  - Modular programming approach for flexibility and scalability.
  - Rigorous testing of the validation and application modules.
- **Date:** 10-11/2023

---

9. Testing

- **Activity:** Conducted unit, integration, and system-level testing.
- **Key Outputs:**
  - Validation of virtual cohorts using real and synthetic datasets.
  - Extensive testing for compliance with Context of Use (CoU) and Question of Interest (QoI) specifications.
  - Identified and resolved programming issues.
- **Date:** 2023-2024

---

10. First Full Prototype with Full Functionality

- **Activity:** Integrated all modules into a fully functional prototype.
- **Key Outputs:**
  - Delivered a working prototype for internal testing and review.
  - Demonstrated full functionality of the validation and application modules.
- **Date:** 10/2023

---

11. First Released Version (to be modified)

- **Activity:** Prepared and released the first public version of the application.
- **Key Outputs:**
  - Documentation completed (README..).
  - Registered on GitHub, Zenodo
  - Pre-version 0.1.0 of the R-statistical environment released.
- **Date:** 11/2023

12. Post-Launch Updates

- **Activity:** Continuous updates and feature enhancements based on feedback.
- **Key Outputs:**
  - Addressed user feedback to improve functionality.
  - Released updates with enhanced statistical methods.
- **Date:** 6/2024

13. Completion and final evaluation of the project SIMCor

- **Activity:** Final review of the application and Github repository.
- **Key Outputs:**
  - Final report of SIMCor
  - Evaluation by the European Commission.
- **Date:** 6 and 9/2024

14. Updates of GitHub

- Activity: **Restructuring and update of the GitHub and registration in ZENODO**
- Key outputs:
  - Restructuring of GitHub
  - Registration at ZENODO
- Date: 1/2025

R-packages integrated in the R-statistical environment

- **Shiny:** create interactive web applications directly in R.
- **Readxl:** read Excel files into R.
- **data.table:** Provides high-performance tools for data manipulation.
- **ggplot2:** Advanced data visualization (scatter plots, boxplots, and heatmaps..)
- **plotly:** Interactive plotting.
- **reshape2:** Reshaping data for analysis (Preparing datasets for multivariate comparisons..)
- **corrplot:** Visualizing correlation matrices (Displaying heatmaps for bivariate correlations)
- **GGally:** Enhancing ggplot2 for advanced visualizations (Pairwise plotting for exploring data relationships.)
- **Boot:** Bootstrap resampling and analysis.
- **Dplyr:** Data manipulation using a consistent grammar.

- **Shinydashboard:** Designing dashboards in Shiny applications.
- **Broom:** Converting statistical models into tidy data frames (Summarizing results for report generation)
- **Survival:** Tools for survival analysis.
- **Pander**
